# An Integrative Genomic Approach to Uncover Molecular Mechanisms of Prokaryotic Traits

**DOI:** 10.1371/journal.pcbi.0020159

**Published:** 2006-11-17

**Authors:** Yang Liu, Jianrong Li, Lee Sam, Chern-Sing Goh, Mark Gerstein, Yves A Lussier

**Affiliations:** 1 Section of Genetic Medicine, Department of Medicine, University of Chicago, Chicago, Illinois, United States of America; 2 Center for Biomedical Informatics, Department of Medicine, University of Chicago, Chicago, Illinois, United States of America; 3 Department of Molecular Biophysics and Biochemistry, Yale University, New Haven, Connecticut, United States of America; 4 Department of Computer Science, Yale University, New Haven, Connecticut, United States of America; 5 Department of Biomedical Informatics, Columbia University, New York, New York, United States of America; University of California Berkeley, United States of America

## Abstract

With mounting availability of genomic and phenotypic databases, data integration and mining become increasingly challenging. While efforts have been put forward to analyze prokaryotic phenotypes, current computational technologies either lack high throughput capacity for genomic scale analysis, or are limited in their capability to integrate and mine data across different scales of biology. Consequently, simultaneous analysis of associations among genomes, phenotypes, and gene functions is prohibited. Here, we developed a high throughput computational approach, and demonstrated for the first time the feasibility of integrating large quantities of prokaryotic phenotypes along with genomic datasets for mining across multiple scales of biology (protein domains, pathways, molecular functions, and cellular processes). Applying this method over 59 fully sequenced prokaryotic species, we identified genetic basis and molecular mechanisms underlying the phenotypes in bacteria. We identified 3,711 significant correlations between 1,499 distinct Pfam and 63 phenotypes, with 2,650 correlations and 1,061 *anti-*correlations. Manual evaluation of a random sample of these significant correlations showed a minimal precision of 30% (95% confidence interval: 20%–42%; *n* = 50). We stratified the most significant 478 predictions and subjected 100 to manual evaluation, of which 60 were corroborated in the literature. We furthermore unveiled 10 significant correlations between phenotypes and KEGG pathways, eight of which were corroborated in the evaluation, and 309 significant correlations between phenotypes and 166 GO concepts evaluated using a random sample (minimal precision = 72%; 95% confidence interval: 60%–80%; *n* = 50). Additionally, we conducted a novel large-scale phenomic visualization analysis to provide insight into the modular nature of common molecular mechanisms spanning multiple biological scales and reused by related phenotypes (metaphenotypes). We propose that this method elucidates which classes of molecular mechanisms are associated with phenotypes or metaphenotypes and holds promise in facilitating a computable systems biology approach to genomic and biomedical research.

## Synopsis

A key challenge of the post-genomic era is to conceive large-scale studies of genomes and observable characteristics of organisms (phenotypes) and to interpret the data thus produced. The goal of this “phenomic” study is to improve our understanding of complex biological systems in terms of their molecular underpinnings. In this paper, Liu and colleagues present comprehensive computational and novel visualization methods for discovering biological knowledge spanning multiple scales of biology. The authors were able to predict and visualize new knowledge between clusters of microbiological phenotypes and their molecular mechanisms. To their knowledge, this is the first time this has been done. More specifically, the method integrates microbiological data with genomic-scale data from protein family databases, gene ontology, and biological pathways. Conducted over 59 fully sequenced bacteria, and including significantly more phenotypes than previous studies of its kind, this study enables a “systems biology” view across different classifications of genes and processes. This represents advancement over previous techniques, which are either limited in biological scale or analytical breadth. Visualization of the networks generated by this technique shows the common biological modules shared by related phenotypes. The results of this experiment demonstrate that the fusion of clinical data with genomic information is able to elucidate, in high throughput, a massive number of biological processes underlying phenotypes.

## Introduction

With the completion of hundreds of prokaryotic genome sequences, computational methods in systems biology aimed at integrating genotypes and phenotypes are developed at an increasing speed. However, data integration and mining remain key challenges in bioinformatics as well as in cross-disciplinary research in biomedical informatics. In addition, a critical issue that remains unsolved is to derive meaningful general biological principles from predictions of statistically significant associations between phenotypes and different biological scales of molecular mechanisms (e.g., protein domains, cellular processes, and cellular pathways) to facilitate the understanding of a particular species. The availability of a large number of fully sequenced genomes and the relatively simple and well-characterized biological processes of prokaryotic organisms makes them ideal model organisms to demonstrate the feasibility of a computational systems biology approach to integrate, mine, and analyze genomic, phenotypic, and functional databases to derive general principles that govern the biology of prokaryotes.

Prokaryotic phenotypes defined by human observations (e.g., motility), living conditions of the organism (e.g., growth at high temperature), and experimental conditions (e.g., acid production in a medium containing D-mannose) are of great interest for post-genomics–era research [1] as well as systems biology research. In clinical microbiological practice, many of these phenotypes are used to discriminate human pathogens from other microorganisms. While a great amount of effort has been devoted to the analysis of prokaryotic phenotypes, prior technologies, operated in a semi-automatic fashion, can at best only analyze a handful of phenotypes at once to deduce their associations with genotypes. In the past, functional genomic approaches predicted prokaryotic genes associated to biochemical pathways [2–4]; however, these studies did not specifically look into systems properties of these genes and were not specifically focused on phenotypes associated to these pathways or the emerging multiscale properties of their molecular mechanisms. Recently, a few studies conducted semi-automatic analyses of associations between an individual prokaryotic phenotype (e.g., hyperthermophily, motility) and its clustering with genes that have similar nucleotide sequences [5] or with Clusters of Orthologuous Groups of proteins (COGs) [5,6]. These studies, limited by their need for manual curation (phenotypic annotations to species), were designed to predict linear relationships between only one biological scale of molecular functions and a limited number of manually annotated phenotypes.

To overcome the limitations of manual annotation in the creation of phenotypic datasets, others in the field conducted phenotype–genotype analyses by mining known knowledge on phenotype–genotype relationships from the scientific literature using high-throughput technologies. In this regard, Korbel et al. used a natural language processing approach to mine the MEDLINE literature and the genomic contents of prokaryotes, resulting in the identification of 2,700 statistically significant associations between COGs and words from the literature related to phenotypes [7]. In other approaches, researchers have built integrated systems to correlate phenotypes, pathways, and genes [3,8]. For example, the WIT system [8] used an integrated system to deduce metabolic systems using genomic data, genes, and pathways. Haft et al*.* built a Web-based system to query and display curated phenotypes and annotated prokaryotic genome properties, such as protein families, pathways, and phylogenies [9]; however, the system does not predict correlations between microbiological phenotypes and genome properties.

In addition to the work being done to integrate prokaryotic phenotypes to genotypes, researchers have also made significant advances in building large-scale phenotype–genotype networks in mice, rats, and humans. The Mouse Genome Database (MGD) has structured their mouse genomic data in terms of the Mammalian Phenotype Ontology [10]. Similarly, the Rat Genome Database (RGD) [11] also developed a phenome database, integrated with its genomic data. In humans, the GeneNetwork (WebQTL) provides a database of complex traits with mappings to quantitative trait loci [12]. And several studies have focused on integrating human phenome and genome resources. For example, Butte et al. created a large-scale phenome–genome network by integrating the Unified Medical Language System with human microarray gene expression data [13]; and Aerts et al. applied a prioritization method to associate genes with human diseases and pathways [14].

We hypothesized that by automatically and simultaneously merging and analyzing massive quantities of microbiological phenotypes and their molecular datasets, we could predict both the molecular underpinnings of prokaryotic phenotypes as well as the relationships between related groups of phenotypes. Thus, this study is designed to illustrate how the big picture emerges from the network of predictions between multiple scales of molecular mechanisms and their correlations to an individual phenotype or to clusters of phenotypes. We developed a high-throughput computational approach, and for the first time, demonstrate the feasibility of integrating a large quantity of prokaryotic phenotypes with genomic datasets from various sources for large-scale data mining across different scales of molecular biology (protein domains, pathways, molecular function, and cellular processes).

To analyze large quantities of prokaryotic phenotypes, we employed the Microbiology module of the Global Infectious Diseases and Epidemiology Network (GIDEON) that we refer to as the Microbiology Knowledge Dataset (MKD) as our source data on phenotypes [15,16]. MKD contains results from laboratory examinations though which users can distinguish different microorganisms. These laboratory results contain descriptions about the morphologic characteristics of microorganisms (e.g., Gram-positive, Gram-negative, motility, and cell wall deficiency), metabolic functions of microorganisms, (e.g., urea hydrolysis, acetate utilization, and gas production from glucose), and microorganisms' adaptation to extreme living conditions, (e.g., growth at 42 °C and growth in 6.5% sodium chloride). We regarded MKD laboratory test results as phenotypes or phenotypic traits, as they constitute observable physical or biochemical characteristics under certain experimental conditions that are determined by the microorganisms' genetic contents. MKD contains more than 100 phenotypic characterizations for more than 3,000 bacterial species, not only allowing us to conduct large-scale data mining on genomics data over phenotypic traits, but also enabling us to compare different phenotypic traits based on their correlations to their genetic contents. Of these 3,000 bacterial species, we included 59 species with fully sequenced genomes in our studies. To integrate phenotypes in MKD with genomic datasets, we chose to include the Protein Family Database (Pfam) [17], Clusters of Orthologous Groups (COGs) [18,19], Kyoto Encyclopedia of Genes and Genomes (KEGG) [20], and biological concepts found in the Gene Ontology (GO) [21,22] which span multiple scales of biology.

Applying our method of data integration and mining, we have identified the genetic basis and molecular mechanisms underlying the many bacterial phenotypes. We revealed 3,711 significant correlations and *anti*-correlations (*p*-value < 0.05) between 63 microbiological phenotypes and 1,499 Pfam families, and identified 17 and 506 significant molecular mechanisms of phenotypes according to our analyses of KEGG's biochemical pathways, and GO's biological concepts, respectively. In addition, for the first time, a novel phenomic analysis was conducted to compare phenotypes with each other on a large scale based on their genetic contents. The original visualization of the network of relationships between one cluster of phenotypes and its significant correlations to protein families, molecular pathways, processes, and function illustrates how clusters of phenotypes (metaphenotypes) share common molecular mechanisms. Such analysis could lead to a better understanding of the molecular relationships between microbial phenotypes on a genomic scale. We believe that this computational technology holds promise in facilitating a systems biology approach to biomedical research in the post-genomic era.

## Results/Discussion

To address our hypothesis, we first describe the results from the high throughput mapping of phenotypes with multiple databases of molecular mechanisms: first the Pfam, followed by KEGG pathways and GO terms. Then, we present results from a combined phenomic analysis of the significant molecular mechanisms across multiple biological scales.

### Mapping the MKD's Clinical Phenotypic Traits to Protein Families

**Figure 1:**
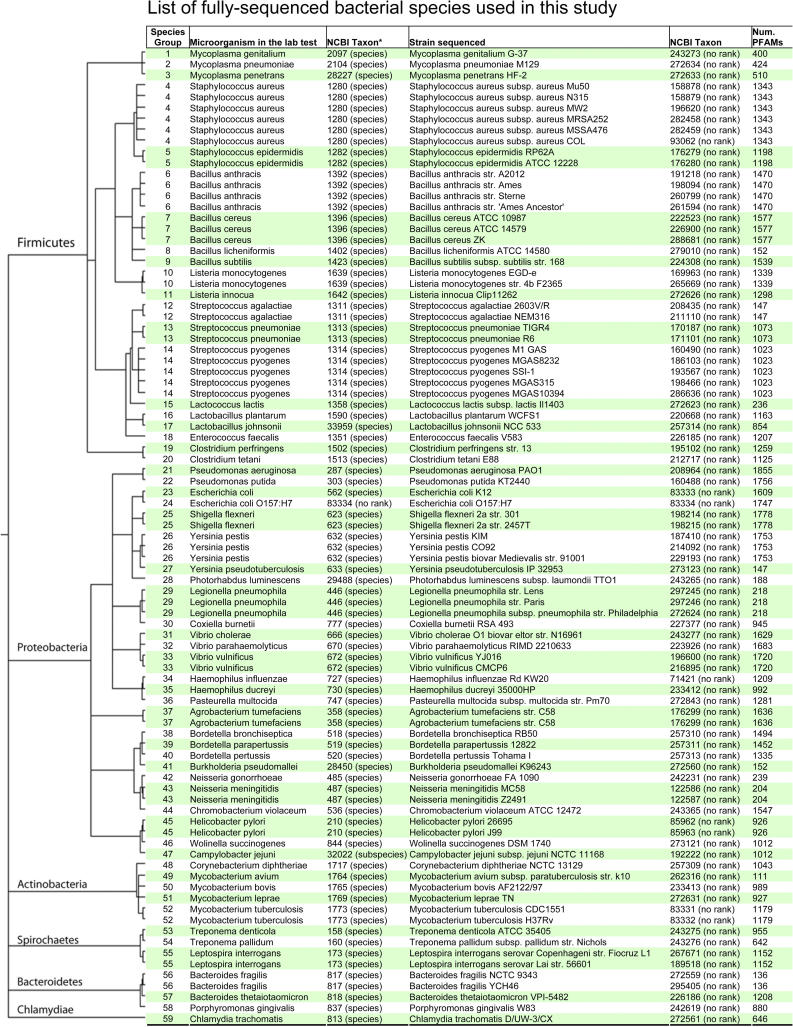
List of Bacterial Species with Full Genome Sequences Used in This Study Bacteria with full genome sequences and laboratory tests used in this study are listed with their phylogenetic tree drawn according to the NCBI taxonomy (the NCBI Taxonomy database is widely used for taxonomy; however, it is not an authoritative source for nomenclature or classification, and an alternate dendogram could have been constructed using the Species2000 Bacteriology Insight Orienting System, http://www-sp2000ao.nies.go.jp/english/bios/). Many of the taxons of species in the laboratory tests are parents of the strains being fully sequenced. The numbers of Pfam families for all species are also shown.

Currently, the availability of more than 208 microbial genome sequences in GenBank provides a rich source of information about the genetic contents of various microorganisms [23]. In addition, functional classification databases, such as COGs [18,19] and Pfam [17], enable us to compare conservation and divergence of functional genes across microorganisms. However, little has been done in the past to correlate the genomic data with phenotypic information. In this study, to uncover the underlying linkages between microorganism phenotypes and their genetic contents, we integrated and analyzed datasets of a microbiological phenotypic database (the MKD) and a genomic protein domains dataset (Pfam). In this study we have, by design, limited the analysis to complete genome sequences to avoid a selection bias toward genes coming from partial genomes that were preferentially sequenced. Methods to deal with organisms with partial genomic sequences will be explored in a future study. As a result, we selected each of the 59 species of microorganisms that exist in both the MKD and Pfam databases with fully deduced genome sequences (Figure 1). These species belong to six phylums, including 20 Firmicutes, 17 Proteobacteria, six Actinobacteria, four Spirochaetes, four Bacteroidetes, and one Chlamydiae, representing about 30% of the bacteria species that have been fully sequenced at the time of this study. Detailed information about their taxonomy in comparison with the fully sequenced bacteria at the time of this study is provided in Table 1. Out of the 208 fully sequenced bacteria available at the time of this study, the 59 species used in this study cover approximately 30% to 40% of available fully sequenced bacterial genomes at different taxonomic levels.

**Table 1:**
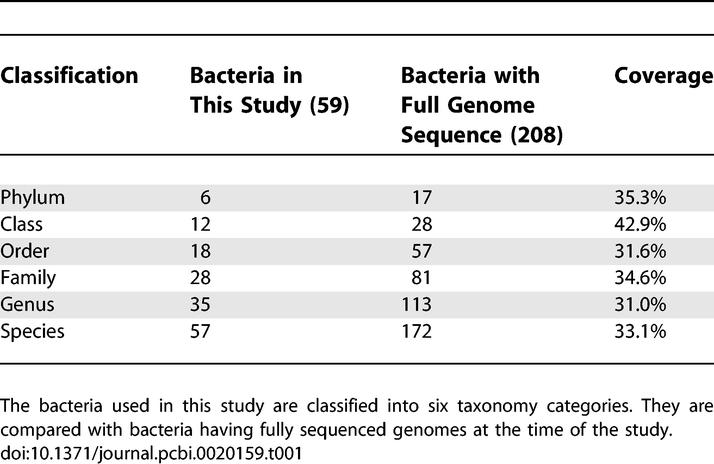
Phylogenetic Classification of Bacteria Used in This Study in Comparison with the Fully Sequenced Bacteria

#### Taxonomical mapping between genomic and phenotypic databases.

The 59 microorganisms were automatically mapped between datasets of MKD and Pfam using the National Center for Biotechnology Information's (NCBI) taxons as a reference, followed by manual examination by experts (Figure 1). Taxons assigned to fully sequenced microorganisms are all at the strain levels at the NCBI (marked as no rank), but those in the MKD are mostly available only at the level of species (57 species, one subspecies, and one no rank). Therefore, most of the organism mappings between MKD and Pfam were either exactly matched or within one taxonomical distance (e.g., a mapping between a species in MKD and a subspecies in fully sequenced bacteria). Given this limitation on data resources, our mapping approach between the phenotypic and genomic datasets is based on the principle that phenotypes for one species are valid for every subsumed strain (one taxonomical range). For example, the MKD contains microbial phenotypes documented as laboratory results for *B. anthracis,* which is defined as a species (Figure 1, Taxon 1392). Four fully sequenced strains of B. anthracis are defined as children of this species and categorized as no rank in the NCBI taxonomy. To control for overrepresentation of a species in the calculation of the hypergeometric distribution, we hence regarded this as a mapping, and all the Pfam families of the four subspecies were merged into one group to compare with the microbial phenotypes of B. anthracis. We took this approach to avoid excluding any Pfam families found in the annotations of the *B. anthracis* species classified as no rank (i.e., in the case that a sequencing error in one strain causes a gene being neglected, protein families associated to this gene would still be included in this study due to the annotation of the other strains). However, this approach also includes some additional Pfam families that belong to horizontally transferred genes from the different strains, which could introduce noise in the study. In addition, a sampling bias may have been introduced in our analysis because the phenotype database pertains to bacteria that are pathogenic or commensal to Homo sapiens and may therefore have more opportunities for horizontal gene transfer than a random set of prokaryotes. In future studies, we intend to combine new weighted statistical approaches that incorporate phylogenetic distance [24] and measurements of horizontal gene transfer [25] with the hypergeometric distribution to control for these potential biases. The number of Pfam families for all bacteria are also shown in Figure 1.

### Identification of Correlations between Bacterial Protein Domains and Phenotypes

**Figure 2:**
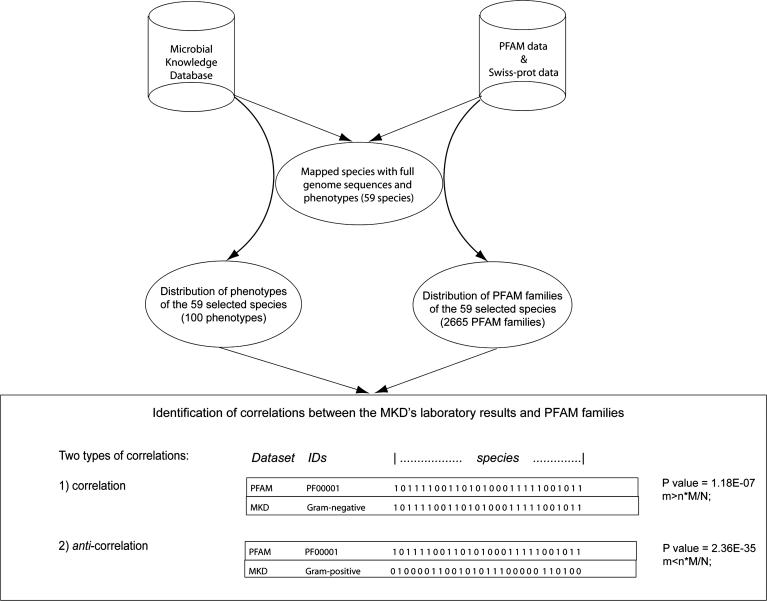
Procedure of Data Integration for Correlating Phenotypes with Pfam Families This flowchart shows how the datasets have been integrated. The calculation of correlations between phenotypes and Pfam families is illustrated in the framed area at the bottom. The formula presented in the box is derived from the hypergeometric distribution and allows for a differentiation between correlation and *anti*-correlation. N, the total number of species used in the study (59); M, the number of species that have a specific Pfam family, such as PF00001 illustrated; n, the number of species that have a specific phenotype, such as Gram-negative; m, the number of species that have both a specific Pfam family and a specific phenotype.

We applied a comprehensive statistical and visualization method based on the hypergeometric distribution to identify the correlations between phenotypic laboratory results and the genetic contents of bacteria. Details are described in Materials and Methods (Equations 1–3), and the procedure is illustrated in Figure 2. In total, we calculated the correlations of the co-occurrences between Pfam families and positive phenotypic laboratory results in the MKD across 59 bacteria species. The correlations can be defined within two categories: 1) correlation, in which the existence of a Pfam family correlates with positive laboratory results; 2) *anti-*correlation, in which existence of a Pfam family correlates with negative phenotypic laboratory results. Two statistical methods were employed to discover correlations and *anti*-correlations: 1) the conservative Šidák adjustment of the *p*-value for multiple a posteriori comparisons [26]; and 2) the calculation of error rates using statistical simulations based on permutation resampling without replacement. These results are available in files available at http://phenos.bsd.uchicago.edu/prok_phenotype/.

Overall, we have identified 3,711 significant correlations between 1,499 distinct Pfam and 63 phenotypes with an experiment-wide error rate of 5%, including 2,650 correlations and 1,061 *anti-*correlations. Here we weight the *anti*-correlations with the same importance as correlations, since the description of the opposite phenotypes would positively correlate with the same set of Pfam families. For example, the phenotype of vancomycin susceptiblity has an *anti*-correlation with the Pfam family of HlyD family secretion protein (PF00529); thus, we can also consider the converse relation to the opposite phenotype: the phenotype of vancomycin resistance has a positive correlation with the same Pfam family. We observed that while some phenotypes (i.e., motility and Gram-negative) correlate with a large number of Pfam families, others correlate with only a few families (i.e., Gelatin hydrolysis and urea hydrolysis). However, the number of correlations inferred by this method depends on both the limitations of the method [5] and the number of available phenotypic laboratory results for different species. For example, Pfam families that exist in all (or no) species would not be correlated with any laboratory results; neither would positive laboratory results that are lacking or existing in most species.

#### Adjusting results for multiple comparisons.

The resulting *p*-values are adjusted for multiple a posteriori comparisons to reduce the experiment-wide error rate. One of the most commonly used methods to control for experiment-wide error rate is the conservative Bonferroni-type adjustment. We used the related Šidák method, as discussed in detail in Materials and Methods (Equation 3), which provides a conservative threshold to filter out false positive results. In this approach, we regarded each phenotypic laboratory test independently, and applied the Šidák adjustment according to the total number of comparisons analyzed. For example, when comparing phenotypes to Pfam families, the number of distinct comparisons with each phenotype is 2,665; the number of proteins that can be compared with each species. Additionally, we limited the comparisons to those phenotypes and Pfam families found in more than three and fewer than 56 species. This resulted in 478 correlations with corrected *p*-values of no more than 0.05 (shown in the file Phenotype_Sidak_Pfam_mapping.xls at http://phenos.bsd.uchicago.edu/prok_phenotype).

Since the Šidák adjustment provides a set of conservative results, many interesting correlations may be consequently filtered out due to its conservative criteria for genome-wide studies, as the variables under study are not entirely independent [27]. In this study, some laboratory tests and the organisms selected are not independent. For example, the laboratory tests Gram-negative and Gram-positive are *anti*-correlated. Organisms are phylogenetically related, of which Firmicutes and Proteobacteria are over-represented in the species used in this study (34% and 29% of the 59 species). Moreover, since the laboratory tests are designed to distinguish bacteria species, there is also a bias on laboratory tests being used to distinguish over-represented species. All of them are currently limitations in this study due to availability of prokaryotic phenotypes limited to MKD—a clinical microbiological database. Certainly, with more species being sequenced and more phenotypic data, we could explore using independent laboratory test results with a set of species more representative of overall prokaryotic diversity.

**Figure 3:**
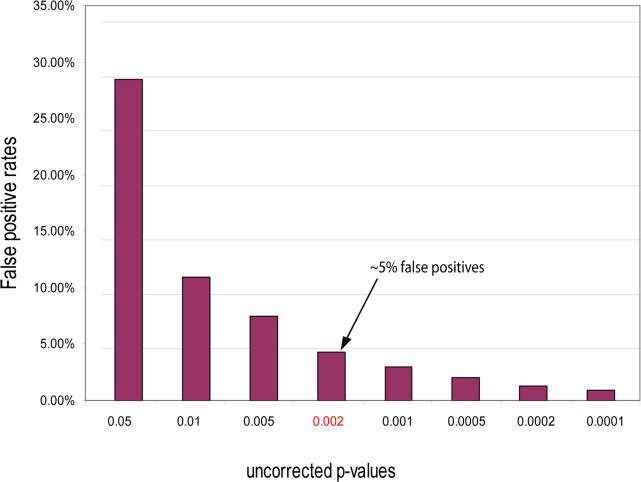
False Positive Error Rates Predicted from Random Datasets According to the Uncorrected Hypergeometric Distribution The false positive error rate represents the ratio of the number of significant correlations from the randomized dataset (control experiment) to the number of significant correlations from the real dataset below a certain *p*-value. At different *p*-value cutoffs, we calculated the error rates from a sample of 1,000 random permutations of the relationship vectors within the dataset (permutation resampling method), and the cutoffs for the highest 1% of occurrences for each uncorrected *p*-value of the hypergeometric distribution (data presented). For uncorrected *p*-values of 0.002 or less, the correlations between phenotypes and Pfam families are predicted to have an error rate of approximately 5%. This cutoff is applied in this study to identify significant correlations.

To overcome these limitations, we applied an additional method based on statistical simulation which can stratify predicted correlations as described in detail in Materials and Methods. With this method, we conducted a permutation resampling in which we compared the number of significant correlations inferred from the original data with those inferred from an experimental control consisting of the distributions of random permutations of the data with different statistical cutoffs for the hypergeometric distribution. Since this method predicts significant correlations in comparison with randomized samples, its results have less stringent cutoffs and cover more phenotypes. Figure 3 summarizes the results from the control experiment over random data, using cutoffs of uncorrected *p*-values, ranging from 0.0001 to 0.05 from the uncorrected hypergeometric test (details in Materials and Methods). As shown in Figure 3, we can expect about 5% of the predictions to be false positives if the uncorrected *p*-value of the hypergeometric distribution is equal to or less than 0.002. In our unadjusted dataset, using an uncorrected *p*-value of 0.002 or less, we identified 3,711 significant correlations in which we expect about 5% to be false positive predictions (data shown in the file Phenotype_Sim_Pfam_mapping.xls at http://phenos.bsd.uchicago.edu/prok_phenotype). By carefully choosing complementary statistical methods for conducting data-mining calculations, we can provide researchers with accurate stratified information on the prediction, yet without filtering out potentially meaningful correlations.

#### Evaluation of the correlations between phenotypes and protein domains.

We conducted an extensive manual evaluation of our predictions, which consists of five parts.

First, we manually examined all the phenotypes (21 phenotypes in total) with their 478 significantly correlated Pfam families based on the Šidák adjustment (data shown in the file Phenotype_Sidak_Pfam_mapping.xls at http://phenos.bsd.uchicago.edu/prok_phenotype). One hundred distinct predictions were manually assessed and 60 were corroborated and annotated with the supporting bibliographic references (Table S2). We then analyzed each of these manually curated sets and provide a summary of the analysis of these predictions for each of the 21 phenotypes (Table S2). Overall, 67% (14) of these phenotypes have at least one Pfam association that was corroborated as shown in Table S2.

Second, we randomly selected 50 positive correlations and 15 *anti*-correlations from the simulation method to evaluate the minimum precision of the predictions. In the evaluation process, we focused on evaluating the positively correlated phenotypes and Pfam families, since *anti*-correlations are often difficult to verify. Of the 50 positive correlations selected, 15 of them were confirmed by supporting literature. As future studies may provide additional corroborations, the precision of 30% (95% confidence interval: 20%–42%; *n* = 50) is a conservative estimate of the overall potential accuracy of the prediction method controlling for false positive rate (also known as false discovery rate) with permutation resampling. Of the 15 *anti*-correlations, two of them (13%) were supported by literature (95% confidence interval: 2%–40%; *n* = 15). A summary of this validation is provided in Table S3 with supportive references for each corroborated prediction.

Third, the false negative rate of the correlations that were regarded as *statistically insignificant* is also estimated. We evaluated 50 random samples, and only one of them has been shown to be correlated, resulting in a 2% false negative rate (95% confidence interval: 0.1%–10%). A summary of this validation is provided in Table S4.

Fourth, we conducted an in-depth evaluation of one phenotype (motility) and compared our results with those from a previously reported study [5], which used a different classification method to cluster full-length genes and interpreted the results using annotations of E. coli genes. We found the results of the two studies to be well-correlated, especially for the top-ranked genes (19 of the top 30, or about 63%, E. coli genes have corresponding Pfam families in the top 30 families in our study: see Table S1).

Fifth, to further evaluate the accuracy of the method in the well-studied phenotype of motility, we performed a manual validation of the predicted results pertaining to bacterial motility mediated through flagella. In this evaluation, significant correlations between phenotypes and Pfam families using the Šidák adjustment and the simulation methods are examined. Since the Šidák adjustment is more conservative, its predicted correlations are also included in those predicted by the simulation method. The results are shown in Table 2, where 18 and 58 Pfam families are predicted by the Šidák and simulation methods, respectively. By manual examination of the annotation of Pfam families, we identified those which participate in bacterial motility, including flagellar mediated motion and chemotaxis. We manually confirmed 12 (out of 18) and 27 (out of 58) Pfam family predictions from the Šidák and permutation resampling methods, respectively. These results confirmed that the Šidák method predicts relatively conservatively, and the data-mining method works equally well to provide accurate predictions. In addition, our results could help improve the functional understanding of current Pfam annotations. For example, we discovered one of the Pfam families, PF06429, described as Domain of unknown function (DUF1078), to be correlated with bacteria motility.

**Table 2:**
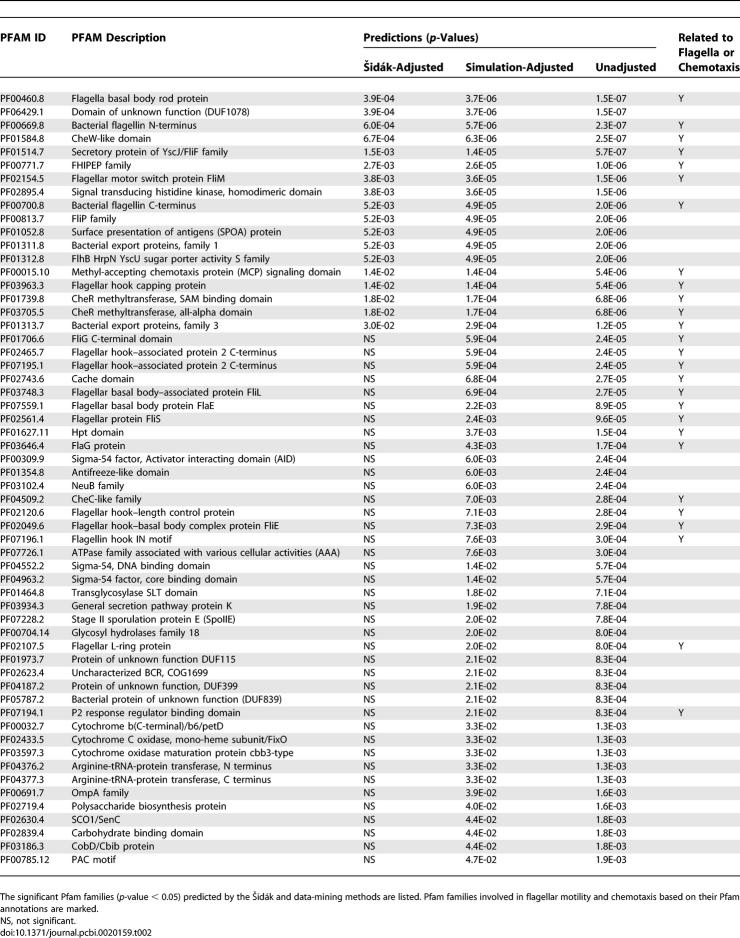
Pfam Families Significantly Correlated with Bacterial Motility

Overall, the results of these evaluations indicate that our approach can faithfully identify the most significantly correlated protein families as accurately as the other classification methods. However, our approach differs from the previous studies because we compared significantly more phenotypes and extended the phenotypic analyses to KEGG pathways and GO concepts which have not previously been analyzed in other studies to our knowledge (discussed below).

#### Limitations of the correlations of phenotypes to protein domain families and future work.

In this study, we primarily used sequence-based classifications (Pfam and COGs) to correlate with phenotypes. The correlations identified by this method suggest hypothetical association based on statistical analysis. However, we limited our exploration of the converse, correlations that are not statistically significant, to the previously described one manual evaluation (Table S5). Though it is feasible to conduct studies to demonstrate that there is not a correlation between certain properties, this was not the design of this study, and therefore we cannot make conclusions about the absence of relationships between correlated elements that did not reach statistical significance in this study. Many factors could lead to statistically insignificant correlations in our approach, for example, the lack of available laboratory data could lead to poor correlations to Pfam families. In future work, it would be interesting to explore the use of structure-based classifications and databases, such as the Structural Classification of Proteins (SCOP) [28], CATH [29], or DALI [30], or using integrated structure and sequence-based classifications, such as classifications based on Pfam domains integrated with Structural Classification of Proteins domains, as studied by Pouliot et al. [31]. Furthermore, we could integrate the classified protein domains with a protein structure database, such as the Protein Data Bank (PDB) [32] or OCA (http://oca.ebi.ac.uk/oca-docs/oca-home.html), to further study their functions.

### Mapping Phenotypes to KEGG Pathways and GO

We also applied the hypergeometric statistical and data-mining approaches to identify correlations of phenotypes with molecular pathways and GO concepts. Using existing bioinformatics resources, we integrated data using the following methods: 1) phenotypes with KEGG molecular pathways by mining their matching COG groups; and 2) phenotypes with GO concepts by mining their matching Pfam families. KEGG pathways and GO concepts significantly correlated with phenotypes were identified by their probabilities of occurrence (see Materials and Methods). This provided more correlations for the mapping, which are likely to reveal biological significance. The details of the procedure are described in Materials and Methods.

We unveiled ten significant correlations and seven significant *anti*-correlations between phenotypes and KEGG pathways and 506 significant correlations between phenotypes and GO concepts. Complete results can be found at the study Web site, http://phenos.bsd.uchicago.edu/prok_phenotype, file Phenotype_KEGG_mapping_results.xls for KEGG mapping and file Phenotype_GO_mapping_results.xls for GO mapping.

Compared with the mapping of phenotypes to Pfam families, which provides the relationships of individual protein domain families to phenotypes, the mapping of phenotypes to GO and pathways provides a systematic view of the underlying molecular mechanisms (from multiple scales of biology) related to phenotypes.

#### Evaluation of the KEGG pathway mappings.

To evaluate the accuracy of our mapping method, we conducted two evaluations: (i) we manually revised each of the 17 predictions, and eight correlations as well as two *anti*-correlations were found corroborated in the literature (Table S5), and (ii) we then pursued a deeper manual evaluation on the most significant mapping results in KEGG. Table 3 shows that two KEGG pathways, the Lipopolysaccharide [33] and Ubiquinone biosynthesis pathways, are significantly correlated with the Gram-negative phenotype, both of which are supported by the literature [34]. In theory, every gene family involved in the Lipopolysaccharide biosynthesis pathway should have significant correlations with the Gram-negative phenotype. Our method accurately identified 15 significantly correlated distinct COGs out of a total of 19 defined in the Lipopolysaccharide biosynthesis pathway. According to the phenotype–COG mapping described in Methods, the remaining four COGs that did not map to the phenotype are COG0438 (predicted glycosyltransferases), COG1442 (Lipopolysaccharide biosynthesis protein: glycosyltransferases), COG0451 (Nucleoside-diphosphate-sugar epimerases), and COG0515 (Serine/threonine protein kinases). This could be due to imprecise definitions in the classification method, resulting in diverse functions of the proteins in the families, as three COGs (COG0438, COG0451, and COG0515) participate in many other pathways; or it could also due to the limitation of our method by using hypergeometric function [5]. In contrast, of the 15 COGs mapped between the Lipopolysaccharide biosynthesis pathway and Gram-negative phenotype, 14 are well-defined and unique to only one pathway, with only one exception (COG0241) that exists in two pathways. This suggests that biases in classification method and gene annotation could reduce the signals for the correlations. Reduction of such biases could improve the accuracy of the prediction of correlations in future studies. Additionally, other data resources could be used in future studies for assessing prokaryotic molecular pathways, such as Metacyc and Ecocyc [35,36].

**Table 3:**
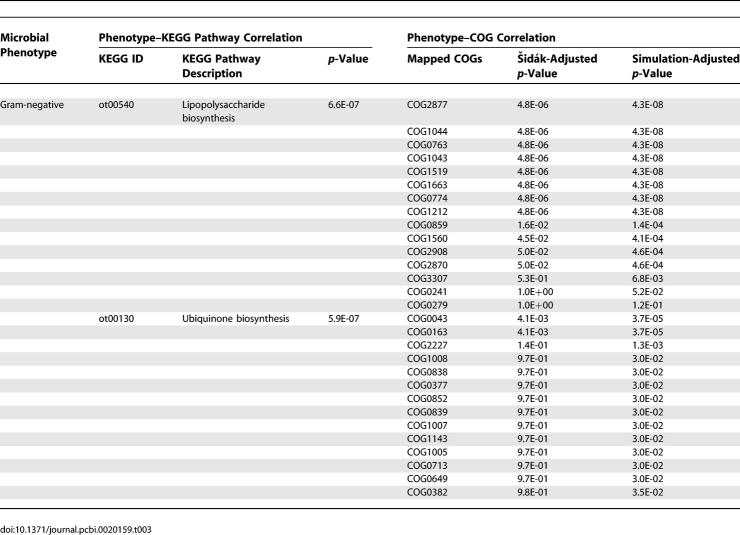
KEGG Pathways and COGs Concepts That Are Significantly Correlated with the Gram-Negative Phenotype

#### Evaluation of GO mappings.

In addition to the phenotype-pathway mapping, we mapped phenotypes to GO concepts (biological processes, molecular functions, cellular components) based on their correlated groups of Pfam families. Using the Šidák adjustment for a posteriori comparisons, there are 309 significant positive correlations with 33 distinct phenotypes within 166 distinct GO terms and 197 *anti*-correlations of 13 unique phenotypes within 142 distinct GO terms. We also provide two evaluations of the GO–phenotype predictions: (i) a random sample of 50 predictions were manually revised and showed a precision of 72% (95% confidence interval: 60%–82%; Table S6), and (ii) a manual evaluation of two phenotypes: Gram-negative and motility. Table 4 shows the GO concepts mapped to the Gram-negative phenotype. Lipopolysaccharide biosynthesis (GO:0009103) and lipid A biosynthesis (GO:0009245) are the top-ranking GO concepts mapped in the biological process branch of GO, while cell (GO:0005623), cell envelope (GO:0030313), and periplasmic space (sensu Gram-negative bacteria) (GO:0030288) are the top-ranking concepts mapped in the cellular component of GO (there are no mappings to the molecular functions of GO). In contrast to the phenotype-pathway mapping, phenotype-GO mapping provides characterizations of phenotypes using different aspects of GO. Though the mappings of phenotypes to pathways and GO concepts were conducted through differently classified gene families (COGs or Pfam), the results are strikingly comparable.

**Table 4:**
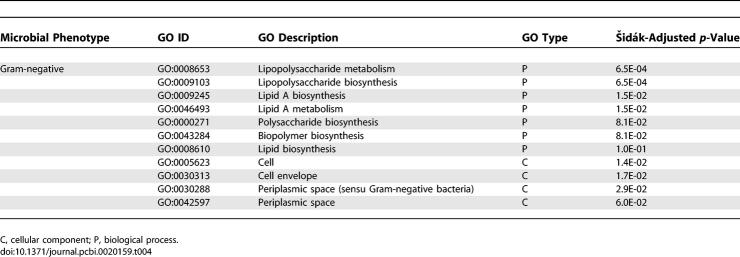
GO Concepts That Are Significantly Correlated with the Gram-Negative Phenotype

By applying a similar mapping to the motility phenotype (Tables 5 and 6), we identified four pathways and 27 GO concepts that are closely correlated with bacterial motility. The three pathways are 1) flagellar assembly, 2) type III secretion system, and 3) bacterial chemotaxis. Bacterial flagellar assembly and chemotaxis pathways are well-known to be important for bacterial motility [37,38], functioning together to guide bacteria's direction of movement. The type III secretion system is known to share many protein structure similarities with the flagellar assembly system in structure, function, and gene sequence [39,40]. Consequently, it is also shown to be significantly correlated with bacterial motility.

**Table 5:**
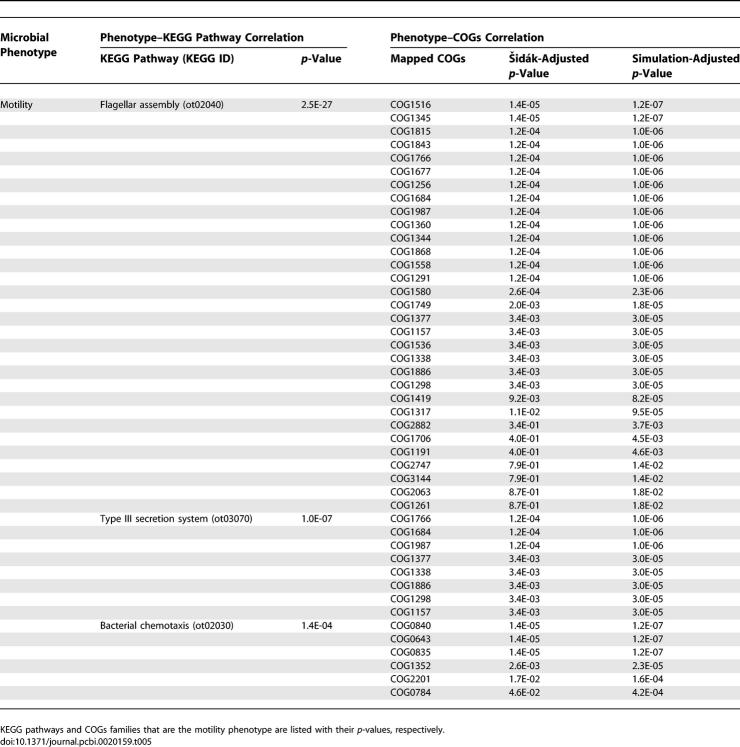
KEGG Pathways and COGs Concepts That Are Significantly Correlated with Bacterial Motility

**Table 6:**
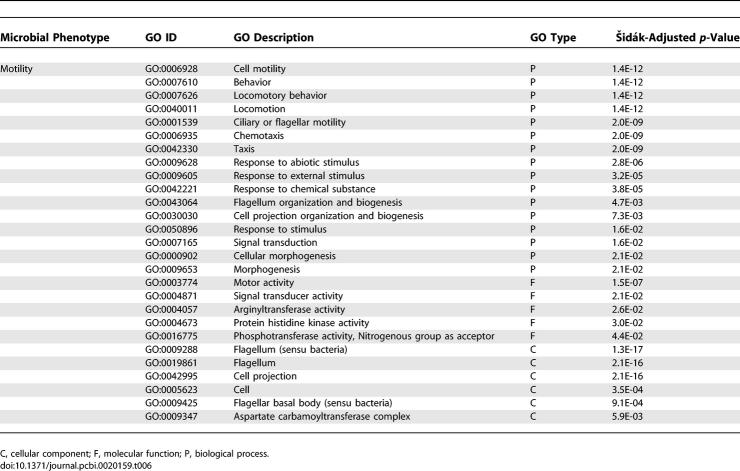
GO Concepts That Are Significantly Correlated with Bacterial Motility

These case studies demonstrate that our high-throughput automated method for mapping phenotypes to pathways and GO concepts can faithfully recapitulate known knowledge. In addition, the method has the potential to predict new correlations between phenotypes and biological systems represented in GO as shown in the complete result datasets at http://phenos.bsd.uchicago.edu/prok_phenotype. While previous correlations studies had been completed on only four phenotypes [5,6], we present an additional 38 phenotype-to-GO correlations. We propose that this method potentially enables a systems-biology approach to analyze genomic datasets by providing a systematic view of the molecular mechanisms beneath phenotypes across different classifications of genes (protein families, pathways, molecular functions, and biological processes). In future studies, we intend to further explore the meaning of directionality of correlations between molecular mechanisms and phenotypes. Indeed, three types of significant correlations can be observed using the hypergeometric distribution: either the observed molecular mechanism is (i) disproportionably associated to a phenotype, or (ii) vice versa, or (ii) both are disproportionably associated to one another.

### Phenomic Analysis and Visualization of Combined Genomic Information across Multiple Biological Scales

**Figure 4:**
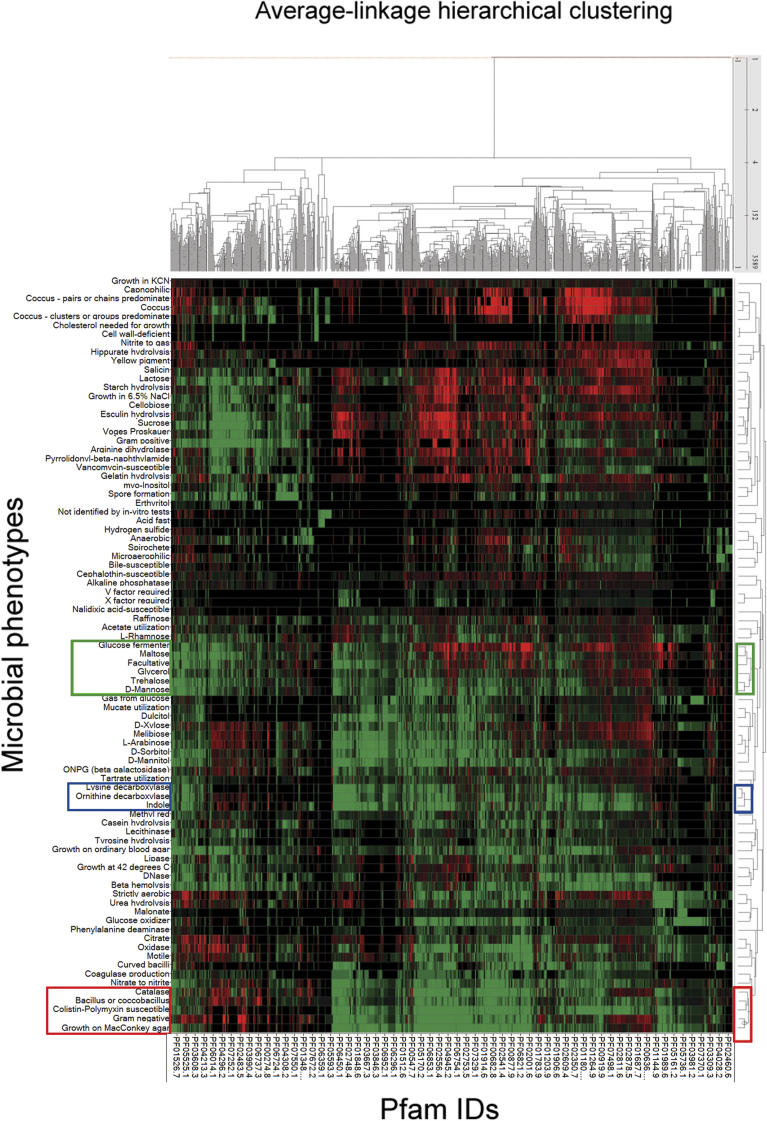
2-D Hierarchical Clustering of Bacterial Phenotypes and Protein Families Phenotypes are on the *x*-axis and Pfam families are on the *y*-axis. Correlation is represented in green, and red represents *anti-*correlation. The three clusters of laboratory tests that are discussed in the paper are highlighted (cluster 1 in a red box; cluster 2 in blue, and cluster 3 in green). We applied continuous coloring representing uncorrected *p*-values from 0 to 10^−4^ (red for *anti-*correlations with the value of −log(*p*-value) for color intensity, and green for correlations with the value of log(*p*-value) for color intensity) for displaying purposes. For details on the hierarchical clustering, see Equation 4 in Materials and Methods.

The results described above systematically provide significant correlations between classes of genes (protein families, pathways, molecular function, and biological processes) and prokaryotic phenotypes. To investigate how information from these classes of genes interacts together on groups of phenotypes, we conducted a cross-phenotype comparison using their correlations to genetic contents. This analysis is anchored on our previously described correlations between prokaryotic phenotypes and Pfam families. All the phenotypes were clustered using a hierarchical average-linkage method based on their correlation scores with Pfam families. Figure 4 shows a 2-D hierarchical clustering of both phenotypes and Pfam families, with green indicating correlation and red indicating *anti-*correlation. To our knowledge, this is the first large-scale cross-phenotype analysis of prokaryotic genomes. We will refer to it as a phenomic analysis, where phenotypes are compared based on their underlying genetic information. Our manual evaluation of two of the largest phenotypic clusters confirmed the results of this automated clustering, showing that biologically relevant phenotypes were generally grouped together. For example, the following phenotypic laboratory tests, Bacillus or coccobacillus, Growth on MacConkey agar, Catalase, Gram-negative, and Colistin-Polymyxin susceptible, are clustered together (highlighted in the red boxes in Figure 4). Within this cluster, the two phenotypes that have the shortest distance to the Gram-negative phenotype, Colistin-Polymyxin susceptible and Growth on MacConkey agar, are known to be closely related to the Gram-negative bacteria. Colistin–Polymyxin is an antibiotic specifically for Gram-negative bacteria [41], and the MacConkey agar test inhibits the growth of Gram-positive bacteria [42]. For the remaining two phenotypes within this cluster (Bacillus or coccobacillus, and Catalase), we were not able to find consistent associations with Gram-negative bacteria from the PubMED database. Gram-positive and Gram-negative prokaryotes are known to have baccillus or cocco-bacillus morphologies, thus the previous correlation could be a bias likely attributable to a disproportionate number of gram-negative species with bacillus morphologies in our dataset. In future studies, we intend to verify whether the same conclusion is generalizable to other bacterial species, and to explore the molecular underpinnings of these relations.

In the second cluster (highlighted in the blue boxes in Figure 4), the following phenotypes were clustered closely: Lysine decarboxylase, Ornithine decarboxylase, and Indole. It is known that ornithine and indole are both involved in amino acid metabolism pathways; ornithine is a derivative of glutamate, and indole is the precursor of tryptophan [43]. Moreover, a protein has been identified in Selenomonas ruminantium that was shown to display the decarboxylating functions of both lysine and ornithine [44]. This is likely because the two functions are essential in this species, thus facilitating such evolution.

**Figure 5:**
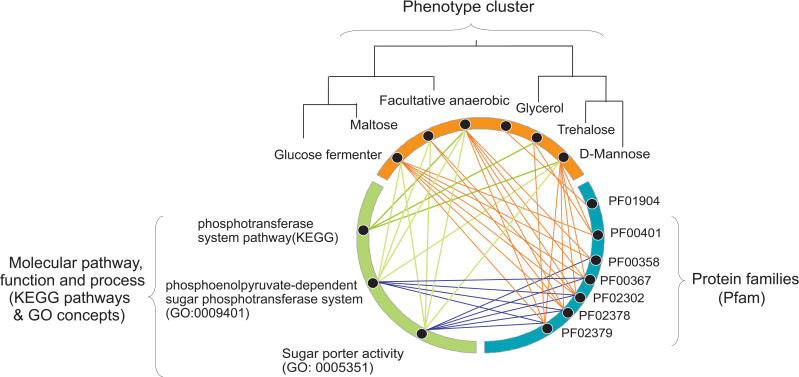
Scalar Network of Correlated Phenotypes, GO, Pathways, and Protein Families As predicted by our study, six phenotypes, taken from a phenotypic cluster in Figure 4 (highlighted there in a green box) are shown highly connected with their significantly correlated biological scales: KEGG pathways, GO concepts, and Pfam families. Every relationship (orange and green lines between concept nodes) has been derived from our study with the exception of relationships between GO and Pfam (blue lines) that were taken from public databases. D-mannose, acid production in a medium containing D-mannose; Facultative anaerobic, facultative anaerobic organism; Glucose fermenter, fermentation in a glucose medium; Glycerol, acid production in a medium containing glycerol; Maltose, acid production in a medium containing maltose; Trehalose, acid production in a medium containing trehalose; PF01904, unknown function; PF00401, ATP Synthase; PF00358, Phosphoenopyruvate-dependent sugar PTS (EIIA 1); PF00367, PTS (EIIB); PF02302, PTS Lactose/Cellobiose specific IIB subunit; PF02378, PTS (EIIC); PF02379, PTS system Fructose-specific IIB subunit.

The third cluster of phenotypes within the green boxes contains six phenotypes related to the catabolism of carbohydrates clustered in the following order: Glucose fermenter (fermentation in a glucose medium), Maltose (production of acid in a medium containing maltose), Facultative anaerobic, Glycerol (production of acid in a medium containing glycerol), Trehalose (production of acid in a medium containing trehalose), and D-mannose (production of acid in a medium containing D-mannose). Every one of these phenotypes is also related to glycolysis [43]. We illustrated this cluster of phenotypes with their significantly correlated Pfam families, GO concepts, and KEGG pathways in detail (shown as a multiscale network in the Figure 5). To constrain the network of *cross*-scale relationships to the most relevant ones, the criteria for displaying a molecular class were the following: 1) GO terms significantly correlated with at least four phenotypes in the cluster, 2) a KEGG pathway with significant correlations to three phenotypes, and 3) Pfam significantly correlated with at least two phenotypes in the cluster (with the exception of one uncharacterized Pfam that has only one link to Glycerol, to illustrate the use of the integrated view for possible predictions). The *cross*-scale relationships between Pfam and GO terms (Figure 5, blue lines) were retrieved from public databases (discussed in Materials and Methods). Using these visualization criteria, we observe that this phenotypic cluster is particularly networked together, as many phenotypes share common KEGG pathways, GO concepts, and Pfam families based on our previous analyses. For example, facultative anaerobic bacteria with ability to metabolize D-mannose share one common KEGG pathway, phosphotransferase system pathway (PTS) and two GO concepts, phosphoenolpyruvate-dependent sugar phosphotransferase system, and sugar porter activity. In addition, three molecular classes obviously related to the carbohydrate transport system in bacteria have been closely associated to the same phenotypic cluster: the KEGG pathway PTS, the cellular process phosphoenolpyruvate-dependent sugar phosphotransferase system PTS, and the molecular function sugar porter activity. Overall, five of the six phenotypes in this cluster share many common protein domain families (Pfam) intervening in the PTS system, as well as higher-level biological concepts, such as GO and KEGG pathways, strongly suggesting similarities or overlaps in their underlying molecular mechanism. In addition to the clustering of phenotypes, clustering of Pfam families based on their correlations to different phenotypes may also provide an informative view of the Pfam families, reflecting their activities in different phenotypes. Macroscopic phenotypes closely related to the catabolism of carbohydrates are thus also highly linked in this illustration with molecular classes closely related to the transport of carbohydrates. This visualization of *cross*-scale relationships, linked together across multiple biological scales and forming a multiscale nexus within the phenomic network, constitutes a proof of concept that the method could be applied to investigate less-understood regions of the network that we developed. We are in the process of further exploring this multiscale network in close collaboration with microbiologists. To our knowledge, this is the first phenomic study designed to predict and visualize *cross*-scale relationships between *clusters* of prokaryotic phenotypes (metaphenotypes) and their molecular mechanisms.

### Conclusion

In this study, we developed a high throughput computational approach capable of automatically *integrating* clinical microbiological laboratory characterizations of bacterial phenotypes with various genomic databases spanning multiple scales of molecular biology (protein domains, pathways, molecular function, and cellular processes). To our knowledge, this is the first study demonstrating the feasibility of integrating a large quantity of prokaryotic phenotypes together with genomic datasets from various sources for large-scale data mining.

Furthermore, in contrast to previous *predictive* studies aimed at building large-scale phenotype–genotype networks, we have thoroughly elucidated systems properties involving multiple scales of molecular mechanisms underlying prokaryotic phenotypes. More specifically, we were able to achieve three objectives. First, we predicted and stratified previously unidentified and uncharacterized correlations (both positive and *anti*- correlations) between protein domain families (Pfam) and bacterial phenotypes using a comprehensive statistical data-mining and visualization method. Our evaluations attest that we faithfully recapitulated known biological knowledge between prokaryotic phenotypes and their molecular underpinnings, demonstrating the validity of our approach to integrate and analyze clinical and genomic datasets. Second, phenotypic information was correlated to additional biological scales such as cellular processes (GO), molecular functions (GO), and molecular pathways (KEGG). Third, the convergence of relationships in the phenomic visualization illustrates the nexus of specific biological systems shared within clusters of related phenotypes (metaphenotypes). This novel phenomic visualization analysis provides insight into the modular nature of common molecular mechanisms spanning multiple biological scales and reused by related phenotypes. We propose that this method, elucidating the relationship between classes of molecular mechanisms and their association with phenotypes or metaphenotypes, holds promise in facilitating a systems biology approach to genomic and biomedical research.

## Materials and Methods

### Datasets.

In this study, we used the following six datasets. 1) Global Infectious Diseases and Epidemiology Network database (http://www.cyinfo.com) [15,16]. It contains an MKD, which contains 100 phenotypic microbiology laboratory results for more than 1,000 microorganism species (92 laboratory tests that contain test results in our 59 selected species were used in this study). The lack of data for some species laboratory tests in the MKD indicates that this knowledge is not useful in clinical bacteriology since MKD has been designed to satisfy the needs of clinical bacteriologists. It does not indicate that the knowledge does not exist elsewhere in the literature. We extracted the MKD data in December 2004. 2) Pfam dataset (release version 16, downloaded in April 2005) [17], of which the Pfam-A classifications were used in this study. 3) KEGG pathway data (KEGG Ontology file (KO), release version 31, downloaded in August 2004) [20,45]. 4) Gene Ontology Annotation (GOA) (downloaded in August 2005) [21,22]. 5) Pfam-GO mapping data, which is maintained by the Gene Ontology Consortium, (downloaded in August 2005 from http://www.geneontology.org/external2go/). 6) COGs data (downloaded in December 2004 from http://www.ncbi.nlm.nih.gov/COG/new/) [19].

### Data integration.

The laboratory results in the MKD are collected for bacterial species, which are primarily used for identifying bacterial strains for medical diagnostics. The MKD rarely has distinct annotations below the taxonomic level of the species according to the NCBI taxonomy. However, bacterial genomes are generally sequenced and annotated at the subspecies or strain levels according to the NCBI taxonomy. A complete list of fully sequenced prokaryotes, many of which have taxonomic annotation (NCBI Taxonomy ID) as no rank at present, was obtained from the NCBI (ftp://ftp.ncbi.nih.gov/genomes/Bacteria/). To map them, we first identified species taxons for the fully sequenced bacteria using the taxonomy tree from NCBI, and then mapped them to the bacteria species in the MKD through computational terminology mapping of text strings [46], followed by manual examination. As a result, we examined nearly 200 bacteria species that have complete genome sequences, and mapped 70 to the bacteria species in the MKD. In the case of a bacterial species having more than one strain with genome sequences, such as *B. anthracis,* all the strains were considered as one species and their genomic data were merged in a lossless way. Of the mapped species, we *merged* the data within each species group (refer to Figure 1 for the 89 fully sequenced genomes organized in species groups) that contained more than 100 Pfam families from the MKD and Pfam databases (altogether 59), and generated a table showing the presence and absence of Pfam families across species as shown in Figure 2. By design, we did not integrate partial genome sequences at the time the mapping started because of the possible bias it might also introduce. In addition, we used the dataset from our prior study [47], which was an integration of the MKD and COGs databases for mapping phenotypes to KEGG pathways.

### Correlating the MKD's clinical diagnostic laboratory data of bacterial phenotypes with functional genomic data.

To investigate whether there are correlations between the clinical diagnostic laboratory results (phenotypes) and the genomic data for bacteria species, we explored the functional classifications of genes. Based on the hypothesis that the existence of a family of genes (or the coexistence of families of genes) is responsible for a phenotype and leads to certain expressed phenotypes under controlled laboratory conditions, we calculated the probability of co-occurrence (by random chance) between a phenotypic laboratory result and presence of a certain cluster (family) of genes across species to uncover such correlations, according to the hypergeometric distribution shown and described below [5].

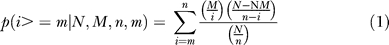



The hypergeometric distribution takes into account the frequencies of species within a specific Pfam to a specific phenotype association and compares it with reference frequencies of species in the entire dataset for (i) the chosen Pfam and (ii) the chosen phenotype, independently of one another. It then calculates the probability (*p*-value) of obtaining these frequencies by chance assuming that the species are randomly distributed across phenotypes and Pfam. A *p*-value smaller than 5%, when corrected for multiple comparisons, indicates that the observed frequency of species sharing a specific Pfam and phenotypes are unlikely to have occurred by chance alone. In our study, there are 59 common species that have diagnostic laboratory results in the MKD and fully sequenced genomes. For instance, the MKD dataset contains 31 (*n*) positive species in the phenotypic class Gram-negative out of 56 species (*N*) for which there are some results for that laboratory data (there is some missing data in MKD because they are not relevant for microbiological characterizations); and the Lipid-A disaccharide synthetase family (Pfam ID: PF2684) has its member domains distributed in 25 (*M*) species. The number of common species between Gram-negative and PF2684 is 24 (*m*). The resulting *p*-value for calculating this co-occurrence distribution by random chance according to the above hypergeometric distribution expression is 1.2 × 10^−8^.

The above-mentioned relationship could have two possible types of correlations: 1) a correlation, referring to a positive laboratory result correlated with the existence of a Pfam family; 2) an *anti-*correlation, referring to a positive lab result that correlates with the absence of a Pfam family. We believe that both correlation types could be equally important for inferring gene functions. To distinguish the two types of correlation, we used the mean value (*μ*) of hypergeometric distribution (shown below) as a reference.

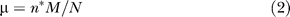



As illustrated in Figure 2, when *m* (Equation 1) is bigger than *μ* (Equation 2), the relationship is a correlation; on the other hand, if *m* is smaller than *μ,* it is an *anti-*correlation. The example above has a mean value of 11.4 (25*32/70), suggesting a correlation. However, if *m* in the above example had been equal to 2, the calculation would show an *anti-*correlation with a *p*-value of 2.5 × 10^−11^.

To control for multiple comparisons, we applied two methods to identify and stratify significant correlations and *anti*-correlations: 1) the conservative Bonferroni-type method known as the Šidák single-step adjusted *p*-value for multiple comparisons [26], and 2) the calculation of error rates using a less conservative data-mining algorithm allowing finding correlation with *p*-value < 0.05.

*Controlling for multiple comparison with a Bonferroni-type method.* The Šidák adjustment for a posteriori comparisons, that was used to maintain an experiment-wide error rate of less than 5%, is calculated according to the following equation,


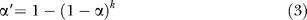


where *α′* and *α* represent the corrected and uncorrected *p*-values, respectively, and *k* represents the number of independent tests. However, since the laboratory dataset contains missing values for some species in different tests, applying the Šidák adjustment for multiple comparisons could be overly conservative or biased toward the laboratory tests with more data.

*Controlling for multiple comparison with a simulation method*. Therefore, to stratify our results with a less conservative method which can predict more correlations, albeit with a higher error rate, we also applied a simulation method to the datasets. Using established statistical resampling principles [48], we created random datasets for a control experiment by generating 1,000 random distributions for each combination of the laboratory results and Pfam families (keeping the total number of occurrences of each lab and Pfam constant in the datasets, while randomizing their distributions in the species—permutation resampling without replacement). For each random distribution, we then calculated the number of statistically correlated laboratory results and Pfam families from these random datasets using the previously described hypergeometric method, with different cutoffs (uncorrected *p*-value <= 0.05, 0.01, 0.005, 0.002, 0.001, 0.0005, and 0.0001). Rather than controlling for multiple comparisons with a statistical test, we used the statistically significant results from the random datasets to predict the number of false positive errors that we should expect in the real dataset when analyzed under the same conditions and subjected to uncorrected multiple comparisons. Since each of the 1,000 random datasets provides a slightly different interpretation using the hypergeometric statistic, we chose a threshold for the calculated hypergeometric statistics that would be observed as the worst case 99% of the time (i.e., 99% confidence). A distribution of the number of errors has been generated for each cutoff, and the numbers that are greater than 99% of the total numbers were selected as references for confidence levels.

*Evaluating the results.* A manual examination was conducted on the predicted results of correlated phenotypes and Pfam families using the two methods. For the Šidák corrected result, we examined the correlated Pfam families for all phenotypes and summarized the results for each phenotype (Table S2). To estimate the false positive rate, we randomly selected 50 predicted phenotype–Pfam correlations from the result of the simulation method, whose Pfam families have biological annotation (i.e., Pfam families annotated as domain with unknown function are not included in this evaluation). Correlations with literature supports were identified as correct predictions from the random set. The false negative rates were also estimated by evaluating a random selection of 50 phenotype–Pfam correlations from all possible combinations between phenotypes and Pfam excluding the significant correlations predicted by the simulated method.

### Correlating MKD's laboratory data with KEGG's molecular pathways and GO concepts.

In a previous study, we calculated correlations between COGs and phenotypes using the hypergeometric and Bonferroni-type methods [47]. In the current study, we also applied the previously described data-mining method to generate a less conservative estimate of phenotypes related to COGs (phenotype–COG dataset), which we have used as intermediary results to compare KEGG's pathways to phenotypes. We also applied the previously described hypergeometric function and Šidák adjustment (Equations 1 and 3) for a posteriori comparisons to identify significant correlations between phenotypes, and either KEGG's molecular pathways or phenotypes and GO concepts. To correlate phenotypes and KEGG molecular pathways, we integrated the correlation of COGs and pathway data from the KEGG ontology file and assigned the following numbers to the hypergeometric function: 1) the number of COGs families in the KEGG ontology file (*N*); 2) the number of correlated COG families for each microbial phenotype from the phenotype–COG dataset (*n*); 3) the number of unique COGs families in each pathway that are also used in this analysis (*M*); 4) the number of common COGs between 2) and 3) (*m*).

To further identify significant correlations between phenotypes and GO concepts, we used a GO term finder software [49] to correlate phenotypes with GO using the Pfam to GO mapping data from the Gene Ontology Consortium. The GO term finder, designed for correlating genes to GO, also exploits the hypergeometric distribution function for identification of significant correlations and provides Šidák-adjusted *p*-values. A set of common Pfam families between the two datasets (Pfam–phenotype and Pfam–GO) was retrieved. Relevant subsets of these two datasets were generated for this study, and subsequently used in calculating phenotypes and GO correlations. The availability of data resources at the time of this study limited our method. Though we first thought to map KEGG through Pfam families, we could not find reliable resources that provide a mapping between them. However, we found a good resource for mapping KEGG and COGs and therefore used it for the study as a convenient alternative.

### Hierarchical clustering of Pfam families and phenotypes for phenomics analysis.

We conducted hierarchical clustering using unweighted average linkage and Euclidean distance of all the phenotypes and Pfam families using normalized correlation *p*-values [50]. The Euclidean distance is defined as:

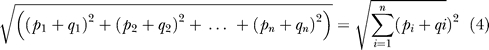
where *P* and *Q* represent series of *p*-values of two phenotypes. To normalize the *p*-values for display purposes, we used the absolute logarithmic value of the *p*-value, and assigned + for positive correlations and − for negative correlations. For example, a *p*-value of 1.0E-07 would be converted to 7 = − (log(1.0E-07)) for positive correlation, and −7 for negative correlation. Therefore, the correlations between Pfam families and phenotypes would be properly represented. We then used the Spotfire software [51] to cluster Pfam families and phenotypes based on the normalized data.

## Supporting Information

Table S1 Comparison of Pfam and E. coli Genes Significantly Correlated to Flagellar-Mediated Motility The top 30 E. coli genes identified by a previous study [5] that correlated with flagellar-mediated motility are compared with their corresponding Pfam families identified in this study. The significance of the correlations defined by the two studies, including uncorrected *p*-values, and *p*-values adjusted according to the Šidák and the resampling methods, are shown.
(85 KB DOC)

Table S2 Evaluation of Phenotypes with Their Significantly Correlated Pfam Families by the Šidák Adjustment Method We manually evaluated 21 phenotypes that have significantly correlated Pfam families by the Šidák adjustment method. Descriptions of the phenotypes are provided with summary and references.
(298 KB DOC)

Table S3 Manual Evaluation of a Random Sample of Correlations and Anti-Correlations of Phenotypes and Pfam Families Fifty positive correlations of phenotype–Pfam were randomly selected from the 3,711 significant correlations by the simulation method. Manual examination indicated that 15 of them have strong literature support (provided), suggesting that they are true positives.
(187 KB DOC)

Table S4 Manual Evaluation of Randomly Selected Correlations, Which Were Statistically Insignificant, to Estimate False Negative Rates A random selection of 50 phenotype–Pfam correlations from all possible combinations between phenotypes and Pfam excluding the significant correlations predicted by the simulated method was evaluated to estimate false negative rate.
(112 KB DOC)

Table S5 Manual Evaluation of Every Statistically Significant Correlation and Anti-Correlation between Phenotypes and KEGG Pathways (68 KB DOC)

Table S6 Manual Evaluation of 50 Randomly Selected Significant Correlations of Phenotype and GO A random selection of 50 phenotype–GO significant correlations was evaluated.
(185 KB DOC)

## Abbreviations

COGs: Clusters of Orthologous Groups
GO: Gene Ontology
KEGG: Kyoto Encyclopedia of Genes and Genomes
MKD: Microbiology Knowledge Dataset from the Global Infectious Diseases and Epidemiology Network database
NCBI: National Center for Biotechnology Information
Pfam: Protein Family Database
PTS: phosphotransferase system pathway
