# Supplementary material for: An Integrative Genomic Approach to Uncover Molecular Mechanisms of Prokaryotic Traits
Source: PLoS Comput Biol. 2006 Nov 17;2(11):e159. doi: 10.1371/journal.pcbi.0020159 (PMC1636675; doi:10.1371/journal.pcbi.0020159)
Supplement: Supplementary file 1 [file pcbi.0020159.st001.doc]

**Supplementary Table 1**. Comparison of PFAM and *E. coli* genes significantly correlated to flagellar mediated motility

| ***E. coli* gene** | **Accession #** | **p-value*** | **Rank*** | **Description** | **Corresponding PFAM families**** | **Šidák adjusted p-value**** |
| --- | --- | --- | --- | --- | --- | --- |
| flgF | P75933 | 3.0E-11 | 1 | +flagellar biosynthesis cell-proximal portion of basal-body rod | PF00460 \| PF06429 | 1.5E-07 \| 1.5E-07 |
| flgG | P0ABW9 | 3.0E-11 | 1 | +flagellar biosynthesis cell-distal portion of basal-body rod | PF00460 \| PF06429 | 1.5E-07 \| 1.5E-07 |
| fliG | P0ABZ1 | 3.0E-11 | 1 | +flagellar biosynthesis, component of motor switch and energizing | PF01706 | 2.4E-05 |
| fhiA | Q47153 | 5.7E-10 | 4 | +polar flagellar assembly protein | PF00771 | 1.0E-06 |
| flhB | P76298 | 5.7E-10 | 4 | +putative part of export apparatus for flagellar proteins | PF01312 | 2.0E-06 |
| fliP | P22586 | 5.7E-10 | 4 | +flagellar biosynthesis protein flip | PF00813 | 2.0E-06 |
| fliF | P25798 | 7.1E-10 | 7 | +flagellar biosynthesis; basal-body membrane ring and collar protein | PF01514 | 5.7E-07 |
| flgC | P0ABX2 | 7.2E-10 | 8 | +flagellar biosynthesis cell-proximal portion of basal-body rod | PF00460 \| PF06429 | 1.5E-07 \| 1.5E-07 |
| fliL | P0ABX8 | 4.6E-09 | 9 | +flagellum-specific ATPase | PF03748 | 2.7E-05 |
| flgE | P75937 | 1.4E-08 | 10 | +flagellar biosynthesis hook protein | PF00460 \| PF06429 \| PF07559 | 1.5E-07 \| 1.5E-07 \| 8.9E-05 |
| fliR | P33135 | 1.4E-08 | 10 | +flagellar biosynthesis | PF01311 | 2.0E-06 |
| cheR | P07364 | 4.5E-08 | 12 | response regulator for chemotaxis; protein glutamate methyltransferase | PF01739 \| PF03705 | 6.8E-06 \| 6.8E-06 |
| fliM | P06974 | 5.6E-08 | 13 | +flagellar biosynthesis component of motor switch | PF02154 | 1.5E-06 |
| cheW | P0A964 | 5.6E-08 | 14 | positive regulator of CheA protein activity | PF01584 | 2.5E-07 |
| flhA | P76298 | 8.6E-08 | 15 | +flagellar biosynthesis; possible export of flagellar proteins | PF00771 | 1.0E-06 |
| flgI | P0A6S3 | 2.2E-07 | 16 | +homolog of Salmonella P-ring of flagella basal body | PF02119 | 5.4E-03 |
| cheB | P07330 | 8.1E-07 | 17 | response regulator for chemotaxis (cheA sensor); protein methylesterase | PF01339 \| PF00072 | 3.3E-03 \| 7.1E-01 |
| cheA | P07363 | 1.1E-06 | 18 | sensory transducer kinase between chemo- signal receptors and CheB and CheY | PF01584 \| PF02895 \| PF01627 \| PF02518 | 2.5E-07 \| 1.5E-06 \| 1.5E-04 \| 1.0E+00 |
| flgH | P0A6S0 | 2.9E-06 | 19 | +flagellar biosynthesis basal-body outer-membrane L ring protein | PF02107 | 8.0E-04 |
| flgK | P33235 | 9.8E-06 | 20 | +flagellar biosynthesis hook-filament junction protein 1 | PF00460 \| PF06429 | 1.5E-07 \| 1.5E-07 |
| motA | P09348 | 3.2E-04 | 21 | +proton conductor component of motor; no effect on switching | NA | NA |
| fliD | P24216 | 9.6E-04 | 22 | +flagellar biosynthesis; filament capping protein; enables filament assembly | PF07195 \| PF02465 \| PF07196 | 2.4E-05 \| 2.4E-05 \| 3.0E-04 |
| mbhA | Q47154 | 9.6E-04 | 22 | * putative motility protein | PF00691 | 1.6E-03 |
| motB | P0AF06 | 9.6E-04 | 22 | +enables flagellar motor rotation, linking torque machinery to cell wall | PF00691 | 1.6E-03 |
| fliN | P0ABZ1 | 2.8E-03 | 25 | +flagellar biosynthesis, component of motor switch and energizing | PF01052 | 2.0E-06 |
| fliS | P26608 | 2.8E-03 | 25 | +flagellar biosynthesis; repressor of class 3a and 3b operons (RflA activity) | PF02561 | 9.6E-05 |
| yahB | P77700 | 2.8E-03 | 25 | putative transcriptional regulator LYSR-type | PF03466\|PF00126 | 3.9E-01 \| 3.9E-01 |
| ydhB | P0ACR2 | 2.8E-03 | 25 | putative transcriptional regulator LYSR-type | PF03466\|PF00126 | 3.9E-01 \| 3.9E-01 |
| tesA | P0ADA1 | 4.2E-03 | 29 | acyl-CoA thioesterase I; also functions as protease I | PF00657 | 3.6E-02 |
| ada | P06134 | 7.9E-03 | 30 | O6-methylguanine-DNA methyltransferase; transcription activator/repressor | PF02805 \| PF01035 \| PF00165 \| PF02870 | 1.2E-02 \| 3.8E-01\| 5.0E-01 \| 2.7E-01 |

\# Accession number of the Uniprot

* p-values and ranks are from previous study (Jim K, Parmar K, Singh M, Tavazoie S (2004) A cross-genomic approach for systematic mapping of phenotypic traits to genes. Genome Res 14: 109-115.)

** p-values and ranks are from this study

NA = Not available
