# Supplementary material for: An Integrative Genomic Approach to Uncover Molecular Mechanisms of Prokaryotic Traits
Source: PLoS Comput Biol. 2006 Nov 17;2(11):e159. doi: 10.1371/journal.pcbi.0020159 (PMC1636675; doi:10.1371/journal.pcbi.0020159)
Supplement: Supplementary file 2 [file pcbi.0020159.st002.doc]

**Supplementary Table 2**. Evaluation of Phenotypes with their significantly correlated Pfam families by the Šidák correction method

| **Phenotype** | **Description** | **Summary of Results** | **Reference (s)** | **Num. correlated Pfam** | **Num. positively correlated Pfam** | **Num. anti-correlated Pfam** | **60 Corroborated Pfams out of a set of 100 reviewed Pfam** |
| --- | --- | --- | --- | --- | --- | --- | --- |
| Anaerobic | Organisms which do not grow in the presence of oxygen | Only one prediction was highly significant according to Bonferroni and also found to be correct in our evaluation: PF02915.6 Rubrerythrin (Rr). According to the PFAM entry, this protein domain is found in anaerobic sulphate-reducing bacteria. According to Karlin et al. “the anaerobic delta genomes encode multiple copies of the anaerobic detoxification protein rubrerythrin that can neutralize hydrogen peroxide.” Additionally, Putz et al have identified rubrerythrin in the anaerobic organelles (hydrogenosomes) of parasitic flagellate Trichomonas vaginalis. | [1,2] | 1 | 1 | 0 | PF02915.6 [3] |
| Bacillus or coccobacillus | Bacterial colony with Bacilli predominate with -highly pleomorphic and inconclusive morphology. | Only one prediction was highly significant according to Bonferroni and also found to be incorrect in our evaluation: PF02388 - FemAB family negatively correlated with bacillus.  According to the PFAM and PubMed entries, this protein domain involved in Staphylococcal species -which are bacilli. | [4,5] | 1 | 0 | 1 |  |
| Catalase | To test for catalase activity, the bacteria to be tested are mixed with Hydrogen Peroxide. Catalase is an enzyme that catalyzes the breakdown of hydrogen peroxide to oxygen and water. | The PFAM family PF00199 (catalase) was perfectly matched to the phenotype associated with catalase activity (p<0.019). The remainder of the predictions could be related to catalase via metabolism (catalytic activity). This is to be expected, as catalase breaks down the cytotoxic hydrogen peroxide byproduct of the electron transport chain. Another group of predictions relate histidine biosynthesis activity with catalase. There is anecdotal evidence that histidine biosynthesis and catalase activity are related in a variety of metabolic processes with over 300 publications in PubMed. | [6] | 15 | 15 | 0 | PF00180.7 [7]  PF00199.8 (#) |
| Coccus | A bacterium having a spherical or spheroidal shape. | Since only negatively correlated Pfam families are identified, which means that the 'coccus' phenotype is associated with the absence of these Pfam families. It suggests that the functions of these Pfam families are not involved in the phenotype. | N/A | 10 | 0 | 10 |  |
| Coccus - pairs or chains predominate | The predominant forms are cocci in chains or pairs. | Since only negatively correlated Pfam families are identified, which means that this phenotype is associated with the absence of these Pfam families. It suggests that the functions of these Pfam families are not involved in the phenotype. | N/A | 6 | 0 | 6 |  |
| Colistin-Polymyxin susceptible | This is a particular susceptibility to the antibiotic colistin (polymyxin E), which are active against gram-negative bacteria. | There are 27 significant Pfam families predicted for this phenotype, and 14 of them are also found in the predicted Pfam families for ‘gram negative,’ such as 'Lipid-A-disaccharide synthetase', and ‘Phosphatidylglycerophosphatase A' that are involved in the lipopolysaccharide biosynthesis. | [8,9] | 27 | 27 | 0 | PF03968.3 [10]  PF04453.4 [10]  PF04972.3 [11] |
| Curved bacilli | Curved, comma or 'gull wing' rods. Does not include helical or spirochete forms | There is only one PFAM family predicted for this phenotype. In PFAM this family includes functionally uncharacterized proteins from such pathogenic bacteria as Helicobacter pylori, Campylobacter jejuni, and Vibrio cholerae, of which all are 'curved bacilli'. | N/A | 1 | 1 | 0 |  |
| D-Mannitol  * Results from this class are explained in more detail in the section **Results and Discussion** | Organisms which grow in the medium containing only D-Mannitol | There are two Pfam families predicted. Both seem to be involved in mannitol metabolism. | [12,13] | 2 | 2 | 0 |  |
| Facultative  * Results from this class are explained in more detail in the section **Results and Discussion** | Organisms which grow both in the presence and absence of air | All the three Pfam families are involved in the phosphotransferase system pathway, which is a major carbohydrate transport system in bacteria. | [14-17] | 3 | 3 | 0 | PF02302.6 [18]  PF00358.7 [18]  PF04215.2 [17] |
| Gram negative * Results from this class are explained in more detail in the section **Results and Discussion** | Gram-negative forms predominate | As discussed in more detail in the paper, the most significant predicted Pfam families for the Gram negative are classified as lipopolysaccharide biosynthesis, lipopolysaccharide metabolism and lipid A biosynthesis, which are characteristics of Gram negative bacteria. | [19-25] | 149 | 86 | 63 | PF04279.4 [26]  PF02321.6 [27]  PF00529.8 [24]  PF00593.10 [28]  PF07715.1 [29]  PF04413.3 [30]  PF07660.2 [31]  PF02684.5 [8]  PF04613.2 [32]  PF03331.3 [33]  PF03279.3 [34]  PF02348.6 [35]  PF06293.3 [36] |
| Gram positive | Gram-positive forms predominate | Compared to the 'Gram positive', the positive correlated Pfam families (86 total) for the 'Gram negative' have a good correlation with the negative correlated Pfam families (24 total) for the 'Gram positive', of which 22 Pfam families are shared. On the other hand, there are 51 shared Pfam families between positively correlated 'Gram positive' Pfam families and negatively correlated 'Gram negative' Pfam families. Some of the top 10 Pfam families are found in the literature to be associated with 'Gram-positive' bacteria. | [15,37] | 110 | 86 | 24 | PF04918.2 [38]  PF04203.3 [39]  PF02684.5 [8]  PF04613.2 [32]  PF03331.3 [40]  PF03279.3 [34]  PF02321.6 [27] |
| Growth on MacConkey agar | MacConkey agar is used for the isolation of gram-negative enteric bacteria and the differentiation of lactose fermenting from lactose non-fermenting gram-negative bacteria. It has also become common to use the media to differentiate bacteria by their abilities to ferment sugars other than lactose. In these cases lactose is replaced in the medium by another sugar. It also inhibits growth of gram-positive bacteria. | Since this test is used to distinguish lactose fermenting gram-negative bacteria, it shares a number of predictions with the 'Gram negative' phenotype. There are 26 positively correlated Pfam families shared between them. 7 out of top 10 most significant correlated Pfam families have unknown function, which may be of interest for further exploration. | [15,37,41] | 111 | 110 | 1 | PF00593.10 [42]  PF07715.1 [29] |
| Growth on ordinary blood agar | This describes a growth phenotype on a general purpose non-selective medium used commonly for cultures. Enriched with whole blood, it is used to isolate certain microorganisms and detect hemolytic activity. | There was only one association with this phenotype and its relationship to the phenotype could not be established in the literature. | N/A | 1 | 1 | 0 |  |
| Indole | Indole is a component of the amino acid tryptophan, which some bacteria are able to metabolize using the enzyme tryptophanase. Indole is tested to determine tryptophan metabolism. | The results associated to this phenotype were in the lower portion of our set near the statistical significance threshold. There was no clear pattern of association between the associated Pfam families and the phenotype. | N/A | 5 | 5 | 0 |  |
| L-Arabinose | This conventional biochemical testing consists on measuring the anaerobic acid production of the bacterial isolate in presence of a medium containing L_Arabinose and no other sugars. | The predicted PFAM family L-arabinose isomerase is a perfect match with the L-Arabinose phenotype. | N/A | 1 | 1 | 0 |  |
| Motile  Results from this class are explained in more detail in the section **Results and Discussion** and in **Tables 4A and 4B** | This phenotype indicates the ability of the organisms to move, typically using flagella or cilia. | The most significant PFAM families associated with this are involved in flagellar motility and chemotaxis. More details are descried in the paper. | [43-59] | 18 | 18 | 0 | PF00460.8 (#)  PF00669.8 (#)  PF01584.8 [60]  PF01514.7 [61]  PF00771.7 [62]  PF02154.5 (#)  PF00700.8 (#)  PF00813.7 [63]  PF01052.8 [64]  PF01311.8 [65]  PF01312.8 [66]  PF00015.10 (#)  PF03963.3 (#)  PF03705.5 [67]  PF01739.8 [67]  PF01313.7 [53] |
| ONPG (beta galactosidase) | ONPG is a lactose analog used to test for beta galactosidase activity. | The PFAM family found to be associated with this phenotype includes beta galacosidase, which facilitates the hydrolysis of ONPG. | [68-73] | 1 | 1 | 0 | PF00703.9 [74] |
| Oxidase | Proteins with oxidase activity participate in a reduction/oxidation reaction where atmospheric oxygen is the electron acceptor | The two Pfam families PF02630.4 and PF00033.8, are a mitochondrion-associated cytochrome c oxidase assembly factor and a component of respiratory chain complex III (which has oxidoreductase activity), respectively. Both are directly associated to oxidase activity. | [75-78] | 2 | 2 | 0 | PF02630.4 [79] |
| Spore formation | This phenotype includes proteins involved in the development of spores. | Except for two Pfam families that have unknown functions, the remaining 9 PFAM families, constituted of proteins involved in spore development as well as structure, are found to be directly related to this phenotype. | [70,80-86] | 11 | 11 | 0 | PF06898.1 (#)  PF03323.3 (#)  PF03419.3 (#)  PF03862.3 (#)  PF05504.1 (#)  PF05580.2 [84]  PF05582.2 [85]  PF07451.1 (#)  PF07454.1 (#) |
| Strictly aerobic | This phenotype describes the ability of organisms to grow only in the presence of air. | Only a single Pfam prediction was found for this phenotype: "Bacterial signaling protein N terminal repeat." According to Galperin et al. it is found exclusively in aerobic or facultative aerobic bacteria. | [87] | 1 | 1 | 0 |  |
| Urea hydrolysis | This phenotype describes an organism’s ability to perform the breakdown of Urea by the enzyme Urease to form ammonia and carbamine acid. | The two Pfam families found significantly associated to this phenotype form some of the subdomains of the Urease enzyme, which is involved in urea hydrolysis. | [88-92] | 2 | 2 | 0 | PF02814.5 (#)  PF05194.2 (#) |

Legend: # denotes self-evident relationships

**References:**
