# Supplementary material for: An Integrative Genomic Approach to Uncover Molecular Mechanisms of Prokaryotic Traits
Source: PLoS Comput Biol. 2006 Nov 17;2(11):e159. doi: 10.1371/journal.pcbi.0020159 (PMC1636675; doi:10.1371/journal.pcbi.0020159)
Supplement: Supplementary file 3 [file pcbi.0020159.st003.doc]

**Supplementary Table 3.** Manual evaluation of 50 randomly selected significantly correlated and 15 significant *anti*-correlated phenotypes and Pfam families

**1) Evaluation of 50 randomly selected positive correlations**

| **Phenotype** | **PFAM** | **Pfam Name** | **Relationship** | **Corroborated** | **Reference** |
| --- | --- | --- | --- | --- | --- |
| Motile | PF00309.9 | Sigma-54 factor, Activator interacting domain (AID) | + | Y | [1] |
| Colistin-Polymyxin susceptible | PF02219.5 | Methylenetetrahydrofolate reductase | + | N |  |
| Motile | PF01584.8 | CheW-like domain | + | Y | [2] |
| Growth on MacConkey agar | PF05068.2 | Mannitol repressor | + | N |  |
| Oxidase | PF03737.4 | Demethylmenaquinone methyltransferase | + | Y | [3] |
| Growth on MacConkey agar | PF00136.9 | DNA polymerase family B | + | N |  |
| Spore formation | PF07451.1 | Stage V sporulation protein AD (SpoVAD) | + | Y | [4] |
| D-Sorbitol | PF06392.1 | Acid shock protein repeat | + | Y | [5] |
| Glucose fermenter | PF01238.9 | Phosphomannose isomerase type I | + | Y | [6] |
| ONPG (beta galactosidase) | PF00703.9 | Glycosyl hydrolases family 2, immunoglobulin-like beta-sandwich domain | + | Y | [7] |
| Glucose oxidizer | PF01322.9 | Cytochrome C' | + | Y | [8] |
| Curved bacilli | PF03445.3 | Putative nucleotidyltransferase DUF294 | + | N |  |
| Ornithine decarboxylase | PF06826.2 | Predicted Permease Membrane Region | + | N |  |
| Growth on MacConkey agar | PF03597.3 | Cytochrome oxidase maturation protein cbb3-type | + | N |  |
| Colistin-Polymyxin susceptible | PF02075.5 | Crossover junction endodeoxyribonuclease RuvC | + | N |  |
| Lysine decarboxylase | PF06029.1 | AlkA N-terminal domain | + | N |  |
| Growth on MacConkey agar | PF03379.3 | CcmB protein | + | N |  |
| Gram positive | PF01316.10 | Arginine repressor, DNA binding domain | + | N |  |
| D-Mannitol | PF01295.8 | Adenylate cyclase, class-I | + | N |  |
| Gram positive | PF07170.1 | Sortase B | + | Y | [9] |
| Catalase | PF03240.3 | FlgA family | + | N |  |
| Facultative | PF04288.3 | MukE-like family | + | N |  |
| Growth on MacConkey agar | PF05690.2 | Thiazole biosynthesis protein ThiG | + | N |  |
| D-Sorbitol | PF04632.2 | Fusaric acid resistance protein conserved region | + | N |  |
| Gram negative | PF00043.12 | Glutathione S-transferase, C-terminal domain | + | N |  |
| Lactose | PF04274.3 | Tagatose 1,6-diphosphate aldolase, (LacD) | + | Y | [10] |
| Gram negative | PF04390.2 | Rare lipoprotein B family | + | N |  |
| Catalase | PF00180.7 | Isocitrate/isopropylmalate dehydrogenase | + | N |  |
| Gram negative | PF03950.5 | tRNA synthetases class I (E and Q), anti-codon binding domain | + | N |  |
| Growth on MacConkey agar | PF00267.9 | Gram-negative porin | + | Y | [11] |
| Sucrose | PF02129.7 | X-Pro dipeptidyl-peptidase (S15 family) | + | N |  |
| Ornithine decarboxylase | PF07361.1 | Cytochrome b562 | + | N |  |
| D-Mannitol | PF04352.3 | ProQ activator of osmoprotectant transporter ProP | + | N |  |
| Growth on MacConkey agar | PF00246.10 | Zinc carboxypeptidase | + | N |  |
| Colistin-Polymyxin susceptible | PF05494.1 | Toluene tolerance, Ttg2 | + | Y | [12] |
| Trehalose | PF00145.7 | C-5 cytosine-specific DNA methylase | + | N |  |
| Growth on MacConkey agar | PF04279.4 | Intracellular septation protein A | + | Y | [13] |
| DNase | PF01316.10 | Arginine repressor, DNA binding domain | + | N |  |
| Coccus | PF06970.1 | Replication initiator protein A (RepA) N-terminus | + | N |  |
| Gram negative | PF00329.7 | Respiratory-chain NADH dehydrogenase, 30 Kd subunit | + | Y | [14] |
| Gram positive | PF00239.10 | Resolvase, N terminal domain | + | N |  |
| Growth on MacConkey agar | PF01212.8 | Beta-eliminating lyase | + | N |  |
| Anaerobic | PF03577.4 | Peptidase family U34 | + | N |  |
| Catalase | PF06481.3 | COX Aromatic Rich Motif | + | N |  |
| D-Mannitol | PF06719.2 | AraC-type transcriptional regulator N-terminus | + | Y | [15] |
| Colistin-Polymyxin susceptible | PF00384.10 | Molybdopterin oxidoreductase | + | N |  |
| Lipase | PF00115.8 | Cytochrome C and Quinol oxidase polypeptide I | + | N |  |
| Growth on MacConkey agar | PF01019.9 | Gamma-glutamyltranspeptidase | + | N |  |
| Oxidase | PF04355.2 | SmpA / OmlA family | + | N |  |
| Colistin-Polymyxin susceptible | PF05235.3 | CHAD domain | + | N |  |

Legend: Relationship + : Statistically significant positive correlation

2) Evaluation of 15 randomly selected anti-correlations

| Coccus - pairs or chains predominate | PF00691.7 | OmpA family | - | N |  |
| --- | --- | --- | --- | --- | --- |
| Coccus | PF02670.4 | 1-deoxy-D-xylulose 5-phosphate reductoisomerase | - | N |  |
| Coccus | PF02749.5 | Quinolinate phosphoribosyl transferase, N-terminal domain | - | N |  |
| Coccus - pairs or chains predominate | PF00015.10 | Methyl-accepting chemotaxis protein (MCP) signaling domain | - | N |  |
| Coccus - pairs or chains predominate | PF02670.4 | 1-deoxy-D-xylulose 5-phosphate reductoisomerase | - | N |  |
| Coccus - pairs or chains predominate | PF02416.5 | mttA/Hcf106 family | - | N |  |
| Starch hydrolysis | PF04413.3 | 3-Deoxy-D-manno-octulosonic-acid transferase (kdotransferase) | - | N |  |
| Esculin hydrolysis | PF04390.2 | Rare lipoprotein B family | - | N |  |
| Coccus - pairs or chains predominate | PF03949.3 | Malic enzyme, NAD binding domain | - | N |  |
| Capnophilic | PF05192.6 | MutS domain III | - | N |  |
| Coccus | PF00892.8 | Integral membrane protein DUF6 | - | N |  |
| Coccus | PF00902.7 | Sec-independent protein translocase protein (TatC) | - | N |  |
| Gram positive | PF02563.5 | Polysaccharide biosynthesis/export protein | - | Y | [16] |
| Strictly aerobic | PF01855.8 | Pyruvate flavodoxin/ferredoxin oxidoreductase, thiamine diP-binding domain | - | Y | [17] |
| Glucose fermenter | PF03524.4 | Conjugal transfer protein | - | N |  |

Legend: Relationship - : Statistically significant negative correlation (*anti*-correlation)

**References:**

1. Jacobi S, Schade R, Heuner K (2004) Characterization of the alternative sigma factor sigma54 and the transcriptional regulator FleQ of Legionella pneumophila, which are both involved in the regulation cascade of flagellar gene expression. J Bacteriol 186: 2540-2547.

2. Sanders DA, Mendez B, Koshland DE, Jr. (1989) Role of the CheW protein in bacterial chemotaxis: overexpression is equivalent to absence. J Bacteriol 171: 6271-6278.

3. Nantapong N, Otofuji A, Migita CT, Adachi O, Toyama H, et al. (2005) Electron transfer ability from NADH to menaquinone and from NADPH to oxygen of type II NADH dehydrogenase of Corynebacterium glutamicum. Biosci Biotechnol Biochem 69: 149-159.

4. Tovar-Rojo F, Chander M, Setlow B, Setlow P (2002) The products of the spoVA operon are involved in dipicolinic acid uptake into developing spores of Bacillus subtilis. J Bacteriol 184: 584-587.

5. Foster JW, Hall HK (1991) Inducible pH homeostasis and the acid tolerance response of Salmonella typhimurium. J Bacteriol 173: 5129-5135.

6. Swan MK, Hansen T, Schonheit P, Davies C (2004) Structural basis for phosphomannose isomerase activity in phosphoglucose isomerase from Pyrobaculum aerophilum: a subtle difference between distantly related enzymes. Biochemistry 43: 14088-14095.

7. Davies G, Henrissat B (1995) Structures and mechanisms of glycosyl hydrolases. Structure 3: 853-859.

8. Moore GR (1991) Bacterial 4-alpha-helical bundle cytochromes. Biochim Biophys Acta 1058: 38-41.

9. Cossart P, Jonquieres R (2000) Sortase, a universal target for therapeutic agents against gram-positive bacteria? Proc Natl Acad Sci U S A 97: 5013-5015.

10. Rosey EL, Oskouian B, Stewart GC (1991) Lactose metabolism by Staphylococcus aureus: characterization of lacABCD, the structural genes of the tagatose 6-phosphate pathway. J Bacteriol 173: 5992-5998.

11. Forbes B, Sahm D, Weissfeld A (2002) Diagnostic Microbiology: Elsevier.

12. Ramos JL, Duque E, Gallegos MT, Godoy P, Ramos-Gonzalez MI, et al. (2002) Mechanisms of solvent tolerance in gram-negative bacteria. Annu Rev Microbiol 56: 743-768.

13. Gilleland HE, Jr., Murray RG (1975) Demonstration of cell division by septation in a variety of gram-negative rods. J Bacteriol 121: 721-725.

14. Soballe K, Overgaard S, Hansen ES, Brokstedt-Rasmussen H, Lind M, et al. (1999) A review of ceramic coatings for implant fixation. J Long Term Eff Med Implants 9: 131-151.

15. Temmerman W, Vereecke D, Dreesen R, Van Montagu M, Holsters M, et al. (2000) Leafy gall formation is controlled by fasR, an AraC-type regulatory gene in Rhodococcus fascians. J Bacteriol 182: 5832-5840.

16. Skurnik M, Bengoechea JA (2003) The biosynthesis and biological role of lipopolysaccharide O-antigens of pathogenic Yersiniae. Carbohydr Res 338: 2521-2529.

17. Chabriere E, Charon MH, Volbeda A, Pieulle L, Hatchikian EC, et al. (1999) Crystal structures of the key anaerobic enzyme pyruvate:ferredoxin oxidoreductase, free and in complex with pyruvate. Nat Struct Biol 6: 182-190.
