# Supplementary material for: An Integrative Genomic Approach to Uncover Molecular Mechanisms of Prokaryotic Traits
Source: PLoS Comput Biol. 2006 Nov 17;2(11):e159. doi: 10.1371/journal.pcbi.0020159 (PMC1636675; doi:10.1371/journal.pcbi.0020159)
Supplement: Supplementary file 4 [file pcbi.0020159.st004.doc]

**Supplementary Table 4.** Manual evaluation of 50 randomly selected correlations, that were statistically insignificant, in order to determine the false negative rate of the predictions.

**Legend: + = statistically insignificant correlation, - =statistically insignificant *anti*-correlation**

| **Phenotypes** | **Pfam** | **Pfam Description** | **P-value (Uncorrected)** | **Type** | **Corroborated?** | **Reference** |
| --- | --- | --- | --- | --- | --- | --- |
| Raffinose | PF01895.6 | PhoU family | 0.46 | - | N |  |
| Yellow pigment | PF02874.7 | ATP synthase alpha/beta family, beta-barrel domain | 0.734 | + | N |  |
| Trehalose | PF03721.3 | UDP-glucose/GDP-mannose dehydrogenase family, NAD binding domain | 0.153 | + | N |  |
| D-Mannitol | PF03571.5 | Peptidase family M49 | 0.583 | - | N |  |
| Lysine decarboxylase | PF01758.6 | Sodium Bile acid symporter family | 0.219 | - | N |  |
| Bacillus or coccobacillus | PF03063.8 | Prismane/CO dehydrogenase family | 0.127 | + | N |  |
| Lactose | PF05239.3 | PRC-barrel domain | 0.189 | - | N |  |
| Raffinose | PF01396.8 | Topoisomerase DNA binding C4 zinc finger | 0.137 | - | N |  |
| Beta hemolysis | PF05480.1 | Staphylococcus haemolytic protein | 0.148 | + | Y | [1,2] |
| Adonitol | PF03739.3 | Predicted permease YjgP/YjgQ family | 1 | - | N |  |
| Oxidase | PF05726.2 | Pirin C-terminal region | 0.018 | + | N |  |
| Growth on MacConkey agar | PF03374.4 | Phage antirepressor protein | 0.305 | - | N |  |
| Coagulase production | PF01396.8 | Topoisomerase DNA binding C4 zinc finger | 0.833 | + | N |  |
| Glucose oxidizer | PF01219.8 | Prokaryotic diacylglycerol kinase | 0.39 | + | N |  |
| L-Arabinose | PF03553.3 | Na+/H+ antiporter family | 0.505 | + | N |  |
| Methyl red | PF04434.5 | SWIM zinc finger | 0.291 | + | N |  |
| Starch hydrolysis | PF05262.1 | Borrelia P83/100 protein | 0.581 | + | N |  |
| Cell wall-deficient | PF04989.2 | Cephalosporin hydroxylase | 0.845 | - | N |  |
| Growth on ordinary blood agar | PF06130.1 | Propanediol utilisation protein PduL | 0.188 | + | N |  |
| Acetate utilization | PF00355.14 | Rieske [2Fe-2S] domain | 0.618 | - | N |  |
| Maltose | PF03119.4 | NAD-dependent DNA ligase C4 zinc finger domain | 0.141 | + | N |  |
| Adonitol | PF05732.1 | Firmicute plasmid replication protein (RepL) | 1 | - | N |  |
| D-Arabitol | PF04093.2 | rod shape-determining protein MreD | 1 | - | N |  |
| D-Xylose | PF07503.1 | HypF finger | 0.4 | - | N |  |
| Lecithinase | PF07186.1 | TraB protein | 1 | - | N |  |
| Growth on ordinary blood agar | PF00902.7 | Sec-independent protein translocase protein (TatC) | 0.176 | + | N |  |
| Capnophilic | PF05651.2 | Putative sugar diacid recognition | 0.04 | - | N |  |
| Alpha-methyl-D-glucoside | PF00296.10 | Luciferase-like monooxygenase | 1 | - | N |  |
| DNase | PF07229.1 | VirE2 | 0.704 | - | N |  |
| Capnophilic | PF03518.3 | Salmonella/Shigella invasin protein B | 0.579 | - | N |  |
| Strictly aerobic | PF03795.3 | YCII-related domain | 0.026 | + | N |  |
| Beta hemolysis | PF02348.6 | Cytidylyltransferase | 0.526 | - | N |  |
| Lactose | PF01208.7 | Uroporphyrinogen decarboxylase (URO-D) | 0.221 | - | N |  |
| Microaerophilic | PF02223.5 | Thymidylate kinase | 0.188 | + | N |  |
| Indole | PF01113.9 | Dihydrodipicolinate reductase, N-terminus | 0.123 | + | N |  |
| Casein hydrolysis | PF01645.6 | Conserved region in glutamate synthase | 0.414 | + | N |  |
| Acetate utilization | PF01420.8 | Type I restriction modification DNA specificity domain | 0.681 | - | N |  |
| Tyrosine hydrolysis | PF04899.2 | MbeD/MobD like | 1 | - | N |  |
| Maltose | PF06315.1 | Isocitrate dehydrogenase kinase/phosphatase (AceK) | 0.411 | - | N |  |
| Acid fast | PF07396.1 | Phosphate-selective porin O and P | 0.929 | - | N |  |
| Tartrate utilization | PF03171.8 | 2OG-Fe(II) oxygenase superfamily | 0.242 | + | N |  |
| Malonate | PF00345.9 | Gram-negative pili assembly chaperone, N-terminal domain | 0.538 | + | N |  |
| Coccus | PF02547.5 | Queuosine biosynthesis protein | 0.031 | - | N |  |
| Sucrose | PF04390.2 | Rare lipoprotein B family | 0.055 | - | N |  |
| Citrate | PF00701.10 | Dihydrodipicolinate synthetase family | 0.6 | + | N |  |
| Adonitol | PF05491.2 | Holliday junction DNA helicase ruvB C-terminus | 1 | - | N |  |
| Hydrogen sulfide | PF01773.8 | Na+ dependent nucleoside transporter N-terminus | 0.148 | - | N |  |
| Phenylalanine deaminase | PF05257.3 | CHAP domain | 0.814 | - | N |  |
| Nitrite to gas | PF02547.5 | Queuosine biosynthesis protein | 0.472 | - | N |  |
| Xanthine hydrolysis | PF01632.6 | Ribosomal protein L35 | 1 | - | N |  |

**References:**

1. Frenette M, Beaudet R, Bisaillon JG, Portelance V (1988) Site of action of a gonococcal growth inhibitor produced by Staphylococcus haemolyticus. J Med Microbiol 26: 199-204.

2. Donvito B, Etienne J, Denoroy L, Greenland T, Benito Y, et al. (1997) Synergistic hemolytic activity of Staphylococcus lugdunensis is mediated by three peptides encoded by a non-agr genetic locus. Infect Immun 65: 95-100.
