# Supplementary material for: An Integrative Genomic Approach to Uncover Molecular Mechanisms of Prokaryotic Traits
Source: PLoS Comput Biol. 2006 Nov 17;2(11):e159. doi: 10.1371/journal.pcbi.0020159 (PMC1636675; doi:10.1371/journal.pcbi.0020159)
Supplement: Supplementary file 5 [file pcbi.0020159.st005.doc]

**Supplementary Table 5**. Manual evaluation of every statistically significant correlation and anti-correlation between phenotypes and KEGG pathways.

| **Microbial phenotype** | **KEGG ID** | **KEGG pathway description** | **Corrected p-value** | **Correlation type** (corr.[+], anti-corr[-]) | **Corroboration in literature** | **Reference** |
| --- | --- | --- | --- | --- | --- | --- |
| Motile | ot02040 | Flagellar assembly | 2.5E-27 | **+** | Y | * |
| Motile | ot03070 | Type III secretion system | 7.4E-09 | **+** | Y | [1] |
| Gram negative | ot00540 | Lipopolysaccharide biosynthesis | 1.9E-06 | **+** | Y | [2,3] |
| Growth on MacConkey agar | ot02040 | Flagellar assembly | 1.1E-05 | **+** | N | - |
| Urea hydrolysis | ot00791 | Atrazine degradation | 3.5E-05 | **+** | N | - |
| Motile | ot02030 | Bacterial chemotaxis | 4.3E-04 | **+** | Y | * |
| Facultative | ot02060 | Phosphotransferase system (PTS) | 3.6E-03 | **+** | Y | [4] |
| Gram negative | ot00130 | Ubiquinone biosynthesis | 6.5E-03 | **+** | Y | [5] |
| D-Mannose | ot02060 | Phosphotransferase system (PTS) | 1.5E-02 | **+** | Y | [6] |
| Trehalose | ot02060 | Phosphotransferase system (PTS) | 3.3E-02 | **+** | Y | [7] |
| Vancomycin-susceptible | ot00540 | Lipopolysaccharide biosynthesis | 2.5E-14 | **-** | Y | [8] |
| Gram positive | ot00540 | Lipopolysaccharide biosynthesis | 1.0E-10 | **-** | Y | [9] |
| Coccus | ot02040 | Flagellar assembly | 1.7E-05 | **-** | N | - |
| Coccus - pairs or chains predominate | ot02040 | Flagellar assembly | 9.5E-05 | **-** | N | - |
| Voges Proskauer | ot02010 | ABC transporters, prokaryotic | 2.7E-02 | **-** | N | - |
| Bacillus or coccobacillus | ot00100 | Biosynthesis of steroids | 3.1E-02 | **-** | N | - |
| Ornithine decarboxylase | ot03014 | Other translation factors | 4.9E-02 | **-** | N | - |

* denotes self-evident relationships

**References:**

1. Gophna U, Ron EZ, Graur D (2003) Bacterial type III secretion systems are ancient and evolved by multiple horizontal-transfer events. Gene 312: 151-163.

2. Nikaido H (1968) Biosynthesis of cell wall lipopolysaccharide in gram-negative enteric bacteria. Adv Enzymol Relat Areas Mol Biol 31: 77-124.

3. Osborn MJ, Rosen SM, Rothfield L, Zeleznick LD, Horecker BL (1964) Lipopolysaccharide of the Gram-Negative Cell Wall. Science 145: 783-789.

4. Postma PW, Lengeler JW, Jacobson GR (1993) Phosphoenolpyruvate:carbohydrate phosphotransferase systems of bacteria. Microbiol Rev 57: 543-594.

5. Meganathan R (2001) Ubiquinone biosynthesis in microorganisms. FEMS Microbiol Lett 203: 131-139.

6. Plumbridge J (1998) Control of the expression of the manXYZ operon in Escherichia coli: Mlc is a negative regulator of the mannose PTS. Mol Microbiol 27: 369-380.

7. Postma PW, Keizer HG, Koolwijk P (1986) Transport of trehalose in Salmonella typhimurium. J Bacteriol 168: 1107-1111.

8. Izaki K, Matsuhashi M, Strominger JL (1968) Biosynthesis of the peptidoglycan of bacterial cell walls. 8. Peptidoglycan transpeptidase and D-alanine carboxypeptidase: penicillin-sensitive enzymatic reaction in strains of Escherichia coli. J Biol Chem 243: 3180-3192.

9. Hogenauer G, Woisetschlager M (1981) A diazaborine derivative inhibits lipopolysaccharide biosynthesis. Nature 293: 662-664.
