# Supplementary material for: An Integrative Genomic Approach to Uncover Molecular Mechanisms of Prokaryotic Traits
Source: PLoS Comput Biol. 2006 Nov 17;2(11):e159. doi: 10.1371/journal.pcbi.0020159 (PMC1636675; doi:10.1371/journal.pcbi.0020159)
Supplement: Supplementary file 6 [file pcbi.0020159.st006.doc]

**Supplementary Table 6.** Manual evaluation of 50 randomly selected significant correlations of phenotype and GO.

| **Microbial Phenotype** | **GO ID** | **GO description** | **GO type** | **Šidák adjusted p-value** | **Correlation type (corr.[+], *anti*-corr[-])** | **Corroboration in literature** | **References** |
| --- | --- | --- | --- | --- | --- | --- | --- |
| Gelatin hydrolysis | GO:0006508 | proteolysis and peptidolysis | P | 0.0393 | + | Y | [1] |
| Colistin-Polymyxin susceptible | GO:0005623 | cell | C | 0.0025 | + | Y | * |
| Cellobiose | GO:0004553 | hydrolase activity, hydrolyzing O-glycosyl compounds | F | 0.0146 | + | Y | [2] |
| Motile | GO:0042221 | response to chemical substance | P | 3.84E-05 | + | Y | [3] |
| Lipase | GO:0009057 | macromolecule catabolism | P | 0.0415 | + | Y | [4] |
| Motile | GO:0042330 | taxis | P | 1.96E-09 | + | Y | * |
| Glucose fermenter | GO:0008643 | carbohydrate transport | P | 1.86E-07 | + | Y | [5,6] |
| Growth on MacConkey agar | GO:0042221 | response to chemical substance | P | 0.0129 | + | Y | [7] |
| Maltose | GO:0051119 | sugar transporter activity | F | 0.0014 | + | Y | [8] |
| Strictly aerobic | GO:0043169 | cation binding | F | 0.0070 | + | Y | [9] |
| Growth on MacConkey agar | GO:0007610 | behavior | P | 1.33E-07 | + | Y | * |
| Oxidase | GO:0042995 | cell projection | C | 0.01216 | + | Y | [10] |
| Gram negative | GO:0030312 | external encapsulating structure | C | 0.0173 | + | Y | [11] |
| Catalase | GO:0016675 | oxidoreductase activity, acting on heme group of donors | F | 0.0308 | + | Y | [12] |
| Urea hydrolysis | GO:0006461 | protein complex assembly | P | 0.0394 | + | Y | [13] |
| Colistin-Polymyxin susceptible | GO:0008104 | protein localization | P | 0.01486 | + | Y | [14] |
| Gram negative | GO:0009245 | lipid A biosynthesis | P | 0.00479 | + | Y | [15] |
| Gram negative | GO:0046493 | lipid A metabolism | P | 0.00479 | + | Y | [15] |
| Facultative | GO:0015290 | electrochemical potential-driven transporter activity | F | 0.0006 | + | Y | [16] |
| Gram negative | GO:0043284 | biopolymer biosynthesis | P | 0.01223 | + | Y | [15] |
| Strictly aerobic | GO:0046914 | transition metal ion binding | F | 0.0490 | + | Y | [17] |
| Urea hydrolysis | GO:0016811 | hydrolase activity, acting on carbon-nitrogen (but not peptide) bonds, in linear amides | F | 0.0038 | + | Y | * |
| Strictly aerobic | GO:0005739 | mitochondrion | C | 0.01896 | + | Y | [18] |
| Glucose fermenter | GO:0015144 | carbohydrate transporter activity | F | 2.56E-08 | + | Y | [5,6] |
| Strictly aerobic | GO:0005740 | mitochondrial membrane | C | 0.0190 | + | Y | [18] |
| Maltose | GO:0008643 | carbohydrate transport | P | 0.0175 | + | Y | [8] |
| Motile | GO:0000902 | cellular morphogenesis | P | 0.0210 | + | Y | [19] |
| Oxidase | GO:0009072 | aromatic amino acid family metabolism | P | 0.0472 | + | Y | [20] |
| Facultative | GO:0008643 | carbohydrate transport | P | 0.0004 | + | Y | [16] |
| Strictly aerobic | GO:0005743 | mitochondrial inner membrane | C | 0.0190 | + | Y | [18] |
| Motile | GO:0043064 | flagellum organization and biogenesis | P | 0.0047 | + | Y | * |
| Motile | GO:0009653 | morphogenesis | P | 0.0210 | + | Y | [19] |
| Glucose fermenter | GO:0005351 | sugar porter activity | F | 2.62E-07 | + | Y | [21] |
| Motile | GO:0004871 | signal transducer activity | F | 0.0208 | + | Y | [22] |
| Strictly aerobic | GO:0043167 | ion binding | F | 0.01890 | + | Y | [17] |
| Catalase | GO:0015002 | heme-copper terminal oxidase activity | F | 0.00268 | + | N | - |
| Beta hemolysis | GO:0008940 | nitrate reductase activity (NADH) | F | 0.0009 | + | N | - |
| DNase | GO:0015294 | solute:cation symporter activity | F | 0.0210 | + | N | - |
| D-Mannitol | GO:0016779 | nucleotidyltransferase activity | F | 0.0304 | + | N | - |
| Colistin-Polymyxin susceptible | GO:0009309 | amine biosynthesis | P | 0.01268 | + | N | - |
| Oxidase | GO:0051234 | establishment of localization | P | 0.0089 | + | N | - |
| Citrate | GO:0016598 | protein arginylation | P | 0.0002 | + | N | - |
| Catalase | GO:0006547 | histidine metabolism | P | 0.0003 | + | N | - |
| Colistin-Polymyxin susceptible | GO:0003774 | motor activity | F | 0.0085 | + | N | - |
| Lipase | GO:0030163 | protein catabolism | P | 0.0099 | + | N | - |
| Colistin-Polymyxin susceptible | GO:0045184 | establishment of protein localization | P | 0.0149 | + | N | - |
| Catalase | GO:0000105 | histidine biosynthesis | P | 1.18E-05 | + | N | - |
| Curved bacilli | GO:0040011 | locomotion | P | 0.0060 | + | N | - |
| Catalase | GO:0006779 | porphyrin biosynthesis | P | 0.01344 | + | N | - |

Legend:

* denotes self-evident relationships

F= Molecular Function, C= Cellular Component, P = Biological Process

**References:**

1. Mc DJ, Weaver RH (1959) Rapid methods for the detection of gelatin hydrolysis. J Bacteriol 77: 60-64.

2. Thompson J, Ruvinov SB, Freedberg DI, Hall BG (1999) Cellobiose-6-phosphate hydrolase (CelF) of Escherichia coli: characterization and assignment to the unusual family 4 of glycosylhydrolases. J Bacteriol 181: 7339-7345.

3. Poole PS, Armitage JP (1988) Motility response of Rhodobacter sphaeroides to chemotactic stimulation. J Bacteriol 170: 5673-5679.

4. James AG, Casey J, Hyliands D, Mycock G (2004) Fatty acid metabolism by cutaneous bacteria and its role in axillary malodour. World Journal of Microbiology & Biotechnology 20: 787-793.

5. Leandro MJ, Goncalves P, Spencer-Martins I (2006) Two glucose/xylose transporter genes from the yeast Candida intermedia: first molecular characterization of a yeast xylose-H+ symporter. Biochemical Journal 395: 543-549.

6. Toivola A, Yarrow D, van den Bosch E, van Dijken JP, Scheffers WA (1984) Alcoholic Fermentation of d-Xylose by Yeasts. Appl Environ Microbiol 47: 1221-1223.

7. Mossel DAA, Scholts HH, Mengerin.Wh (1962) Use of a Modified Macconkey Agar Medium for Selective Growth and Enumeration of Enterobacteriaceae. Journal of Bacteriology 84: 381-&.

8. Jones CR, Ray M, Dawson KA, Strobel HJ (2000) High-affinity maltose binding and transport by the thermophilic anaerobe Thermoanaerobacter ethanolicus 39E. Applied and Environmental Microbiology 66: 995-1000.

9. Chen XH, Christopher A, Jones JP, Bell SG, Guo Q, et al. (2002) Crystal structure of the F87W/Y96F/V247L mutant of cytochrome P-450cam with 1,3,5-trichlorobenzene bound and further protein engineering for the oxidation of pentachlorobenzene and hexachlorobenzene. Journal of Biological Chemistry 277: 37519-37526.

10. Gilardi GL (1967) Morphological and Biochemical Characteristics of Aeromonas Punctata (Hydrophila Liquiefaciens) Isolated from Human Sources. Applied Microbiology 15: 417-&.

11. Beveridge TJ (1999) Structures of gram-negative cell walls and their derived membrane vesicles. Journal of Bacteriology 181: 4725-4733.

12. Baker RD, Cook CO, Goodwin DC (2006) Catalase-peroxidase active site restructuring by a distant and "inactive" domain. Biochemistry 45: 7113-7121.

13. Park JU, Song JY, Kwon YC, Chung MJ, Jun JS, et al. (2005) Effect of the urease accessory genes on activation of the Helicobacter pylori urease apoprotein. Mol Cells 20: 371-377.

14. David HL, Rastogi N (1985) Antibacterial action of colistin (polymyxin E) against Mycobacterium aurum. Antimicrob Agents Chemother 27: 701-707.

15. Wu T, McCandlish AC, Gronenberg LS, Chng SS, Silhavy TJ, et al. (2006) Identification of a protein complex that assembles lipopolysaccharide in the outer membrane of Escherichia coli. Proc Natl Acad Sci U S A 103: 11754-11759.

16. Boekhorst J, Siezen RJ, Zwahlen MC, Vilanova D, Pridmore RD, et al. (2004) The complete genomes of Lactobacillus plantarum and Lactobacillus johnsonii reveal extensive differences in chromosome organization and gene content. Microbiology 150: 3601-3611.

17. Alexeev D, Zhu H, Guo M, Zhong W, Hunter DJ, et al. (2003) A novel protein-mineral interface. Nat Struct Biol 10: 297-302.

18. Brandt U, Abdrakhmanova A, Zickermann V, Galkin A, Drose S, et al. (2005) Structure-function relationships in mitochondrial complex I of the strictly aerobic yeast Yarrowia lipolytica. Biochem Soc Trans 33: 840-844.

19. Doino JA, McFallNgai MJ (1995) A transient exposure to symbiosis-competent bacteria induces light organ morphogenesis in the host squid. Biological Bulletin 189: 347-355.

20. Li J, Osyczka A, Conover RC, Johnson MK, Qin H, et al. (2003) Role of acidic and aromatic amino acids in Rhodobacter capsulatus cytochrome c1. A site-directed mutagenesis study. Biochemistry 42: 8818-8830.

21. Heiland S, Radovanovic N, Hofer M, Winderickx J, Lichtenberg H (2000) Multiple hexose transporters of Schizosaccharomyces pombe. J Bacteriol 182: 2153-2162.

22. Lang AS, Beatty JT (2002) A bacterial signal transduction system controls genetic exchange and motility. J Bacteriol 184: 913-918.
